# Interplay of histidine residues of the Alzheimer’s disease Aβ peptide governs its Zn-induced oligomerization

**DOI:** 10.1038/srep21734

**Published:** 2016-02-22

**Authors:** Andrey N. Istrate, Sergey A. Kozin, Sergey S. Zhokhov, Alexey B. Mantsyzov, Olga I. Kechko, Annalisa Pastore, Alexander A. Makarov, Vladimir I. Polshakov

**Affiliations:** 1 Engelhardt Institute of Molecular Biology, Russian Academy of Sciences, 119991, Moscow, Russia; 2 Faculty of Fundamental Medicine, M.V. Lomonosov Moscow State University, 119991, Moscow, Russia; 3 The Wohl Institute, King’s College London, SE5 9RT, London, UK

## Abstract

Conformational changes of Aβ peptide result in its transformation from native monomeric state to the toxic soluble dimers, oligomers and insoluble aggregates that are hallmarks of Alzheimer’s disease (AD). Interactions of zinc ions with Aβ are mediated by the N-terminal Aβ_1–16_ domain and appear to play a key role in AD progression. There is a range of results indicating that these interactions trigger the Aβ plaque formation. We have determined structure and functional characteristics of the metal binding domains derived from several Aβ variants and found that their zinc-induced oligomerization is governed by conformational changes in the minimal zinc binding site _6_HDSGYEVHH_14_. The residue H6 and segment _11_EVHH_14_, which are part of this site are crucial for formation of the two zinc-mediated interaction interfaces in Aβ. These structural determinants can be considered as promising targets for rational design of the AD-modifying drugs aimed at blocking pathological Aβ aggregation.

According to the amyloid hypothesis, which has been the predominant framework for Alzheimer disease (AD) studies, Aβ aggregation has a unique and critical role as an initiator of AD pathology^1^,^2^. What triggers Aβ aggregation still remains unclear, however, some genetically and/or post-translationally modified Aβ species accumulated in the amyloid plaques appear to act as the pathogenic aggregation seeds^3^. It has been shown in animal models of AD that zinc ions might play a crucial role in the Aβ plaque formation *in vivo*^4^,^5^,^6^,^7^. Indeed, at concentration as high as that detected in the synapse, zinc ions specifically bind Aβ and are able to facilitate Aβ aggregation^8^, which could explain abnormally high levels of zinc ions within amyloid plaques of AD patients^9^,^10^. It is assumed that zinc ions promote Aβ aggregation via population shift of polymorphic states^11^ with a mechanism similar to that observed for larger zinc-binding proteins^12^. When zinc-induced Aβ amyloidogenesis is observed *in vitro,* conformational changes in Aβ are also identified^13^. However, precise structural details of these changes were elusive since three-dimensional structures of Aβ oligomers complexed with zinc ions were unavailable^14^.

Interaction of Zn^2+^ with monomeric Aβ species is mediated by the metal binding domain which comprises the N-terminal region 1–16 of Aβ^15^,^16^,^17^. Aβ_1–16_ exists in health and disease as a separate entity^18^, suggesting its possible role as the structural and functional unit of the full-length Aβ. Indeed, the interaction of N-terminal region 1–16 with the β-strand hydrophobic region 17- 42 is negligible in the model amyloid aggregates^19^,^20^, and also synthetic peptides Aβ_1–16_ exhaustively simulate the metal binding properties of Aβ^15^,^16^,^21^. Previous NMR studies of the N-terminus of Aβ showed that the first 9 residues are poorly structured, whereas residues beyond 10 form a distinct local conformation^17^,^22^,^23^,^24^,^25^,^26^,^27^,^28^,^29^,^30^. Structure of the tethered N-terminus of the Alzheimer’s disease amyloid-β peptide obtained using X-ray crystallography^31^ showed that Aβ region 10–16 is relatively rigid and adopts a mixture of the local polyproline II-helix (PPII) and turn type conformations. The fragment Aβ_1–16_ includes several charged residues with their location typical of ionic self-complementary peptides^32^. These residues participate in the formation of electrostatic contacts, which can stabilize both intra and intermolecular interactions. The region 10–16 of Aβ appeared to be an effective metal ion trapping unit^33^. The fragment 6–14 of Aβ has been determined as the minimal Zn^2+^ binding site wherein the ion is coordinated by H6, E11, H13, and H14^34^. Under physiological conditions in the presence of Zn^2+^ the metal binding domains of several natural Aβ variants form homo- and hetero-dimeric complexes^35^,^36^,^37^,^38^. Residues 11–14 of the two interacting subunits compose the dimer interface wherein two pairs of E11 and H14 residues coordinate a zinc ion^36^,^38^.

Along with the intact Aβ isoforms which are heterogenous at their N-termini and/or C-termini, the amyloid plaques involve a variety of chemically modified Aβ variants^39^. The Aβ species extracted from the plaques can initiate pathological aggregation of endogenous Aβ upon intracerebral injections into animal models of AD^3^,^40^,^41^. Several post-translational modifications have been discovered to increase the aggregation rate of Aβ^42^. Some chemical modifications and amino acid changes within the metal binding domain of Aβ (e.g. isomerization of D7, phosphorylation of S8, and the H6R English familial mutation associated with early onset AD) facilitate zinc-dependent dimerization and/or oligomerization of the domain^36^,^38^,^43^, thus suggesting their potential role in initiating the pathological aggregation process. Indeed, peripheral injections of the synthetic Aβ species bearing isomerized D7 (isoD7-Aβ) was shown to trigger cerebral amyloidosis *in vivo*^44^. Little is known about the Aβ metal binding sites in the aggregated state (oligomers or fibrils), but in general, it seems that the binding sites are similar to those in the monomeric peptide^11^,^45^.

In the current study, we have used high resolution NMR spectroscopy to reveal the structural determinants of zinc-induced Aβ oligomerization, i.e. the structure elements that are responsible for the ability of Aβ to form zinc-bound intermolecular complexes. Synthetic peptides corresponding to the metal binding domains of the intact Aβ (Aβ_1–16_), of the English H6R Aβ mutant (H6R-Aβ_1–16_), Aβ containing isomerized D7 (isoD7-Aβ_1–16_) and several truncated and mutant forms of these peptides have been used as experimental models (Fig. 1). Solution structure of the zinc-bridged H6R-Aβ_1–16_ dimer has been determined. This structure provides direct information on the primary zinc-mediated Aβ dimer interface, formed by the residues ^11^EVHH^14^. The role of residues H6 and H13 in the emergence of the second Zn^2+^-dependent interface within the interacting metal binding domains Aβ_1–16_ and isoD7-Aβ_1–16_ during their zinc-induced oligomerization has been also revealed. Taken together, the data indicate that interplay of histidine residues in the minimal zinc-binding site 6–14 of Aβ upon its interactions with zinc ions underlies critical conformational changes of Aβ, which in turn lead to Aβ dimerization, oligomerization and aggregation. The results provide structural basis for rational design of the AD-modifying drugs aimed at blocking pathological Aβ aggregation.

## Results

### NMR signal assignments

Earlier we reported chemical shift assignments of Аβ_1–16_^17^ and H6R-Аβ_1–16_^36^. ^1^H, ^13^C and ^15^N chemical shifts of the peptides isoD7-Аβ_1–16_, isoD7-Аβ_1–10_, isoD7-H13R-Аβ_1–16_, Аβ_6–16_ and their complexes with Zn^2+^ ions have been assigned by analysis of the set of 2D homonuclear (NOESY, ROESY, TOCSY, DQF-COSY) and heteronuclear (^13^C-^1^H HSQC and ^15^N-^1^H HSQC) NMR experiments. Heteronuclear spectra (Figs S1–S4) have been acquired at the natural abundance of ^13^C and ^15^N isotopes. In the case of isoD7-Аβ_1–16_, isoD7-H13R-Аβ_1–16_ and Аβ_6–16_^15^N resonance assignments have been obtained for the free peptides only, as substantial zinc-induced signal broadening does not allow collection of heteronuclear correlation spectra at the natural abundance of ^15^N. Resonance assignments for nearly all ^1^H and ^13^C nuclei have been determined for all studied peptides and their zinc complexes (Tables S1–S7).

### Structure of the metal binding domains Аβ_1–16_, H6R-Аβ_1–16_ and isoD7-Аβ_1–16_ in free state in solution

**Figure 2:**
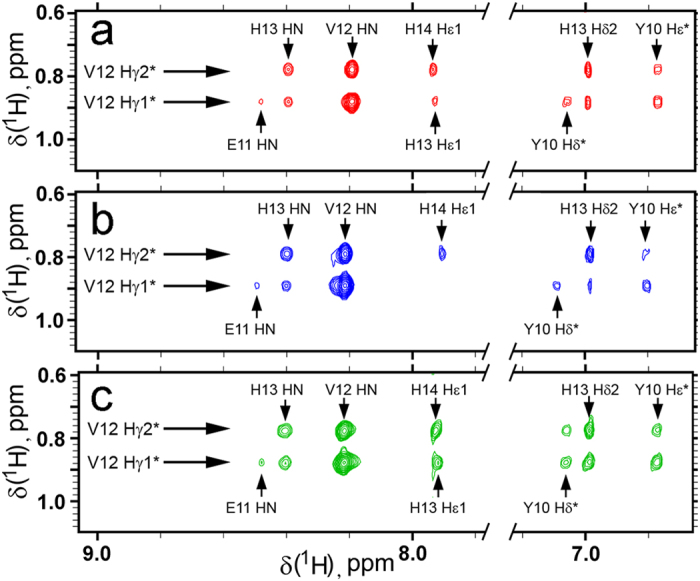
Fragments of NOESY spectra (mixing time 250 ms) of free peptides (**a**) Аβ_1–16_, (**b**) H6R-Аβ_1–16_, and (**c**) isoD7-Аβ_1–16_ illustrating similar patterns of sequential and medium-range NOEs with the participation of the methyl groups of V12. Spectra were recorded in 90% H_2_O/10% D_2_O, in presence of 10 mM bis-tris-d_19_, pH 6.8, at 283K.

Structures of the peptides Аβ_1–16_, H6R-Аβ_1–16_ and isoD7-Аβ_1–16_ in free state in solution is virtually identical, as shown by the NMR spectra of the peptides measured in the absence of zinc ions (Fig. 2, Figs S5 and S6). Despite the fact that the peptides are relatively flexible, as evidenced by their narrow lines in NMR spectra, several distinct cross peaks detected in NOESY spectra indicate that the dominant backbone conformation in the region of residues 10–15 is close to the left-handed polyproline-II helix, observed in the crystal structure of Аβ_1–16_^31^. NOEs between amide protons of neighboring residues HN^i^-HN^i−1^ and between amide protons and Hα (HN^i^-Hα^i−1^, HN^i^-Hα^i−2^, HN^i^-Hα^i+1^, Figs S5 and S6) confirm such dominant conformation of the fragment 10–15. Proximity of methyl groups of the residue V12 to the side chains of Y10 and H14 (Fig. 2), observed in all three investigated peptides should also be noted. Thus, the fragment 10–15 in all three peptides has the same dominant structure, which can determine zinc-trapping properties of the metal binding domain.

### Aggregation states of H6R-Аβ_1–16_, isoD7-Аβ_1–16_ and Аβ_6–16_ in the presence of zinc ions

The peptides behave differently in the presence of Zn^2+^. Addition of equimolar amounts of zinc ions to the peptides isoD7-Аβ_1–16_, Аβ_6–16_ and Аβ_1–16_ resulted in precipitation of the peptides in contrast to H6R-Аβ_1–16_, which remained in solution in the studied concentration range (up to ~10 mM). These observations show that isoD7-Аβ_1–16_, Аβ_6–16_ and Аβ_1–16_ undergo zinc-induced oligo- and/or polymerization. The precipitated peptides dissolve in acidic conditions (pH<4), but precipitate again upon the increase of pH above ~6. Such pH dependence indicates involvement of histidine residues in zinc binding. Precipitation of isoD7-Аβ_1–16_ has been observed at peptide concentration above ~0.3 mM, Аβ_6–16_ above ~0.8 mM and Аβ_1–16_ above ~5 mM (Table 1). DLS studies evidence the presence of soluble zinc-induced oligomers during precipitation of the peptides (Table 1). These data indicate that soluble zinc-induced oligomers precede insoluble aggregates. NMR studies of the interaction of isoD7-Аβ_1–16_ and Аβ_6–16_ with Zn^2+^ ions have been carried out at concentration below ~0.3 mM, where both peptides remain soluble.

**Figure 3:**
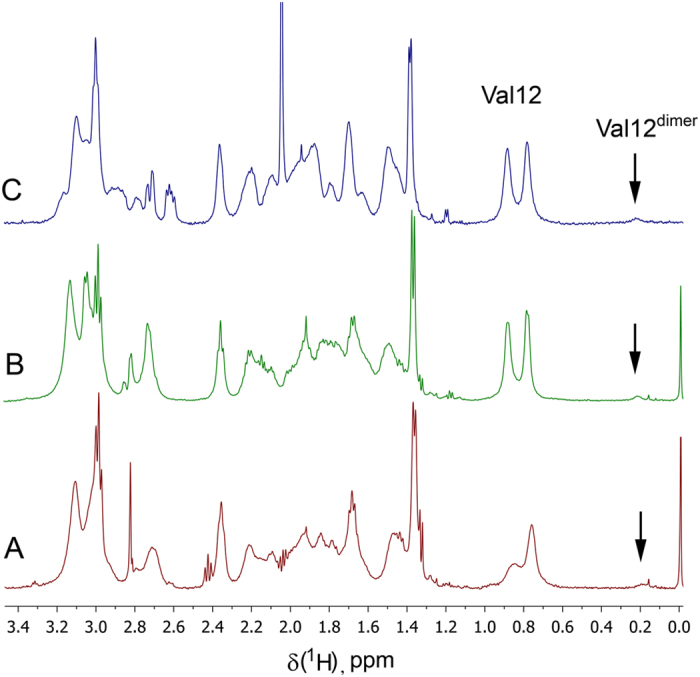
Fragments of 1D spectra of Аβ_1–16_ (**A**), H6R-Аβ_1–16_ (**B**), and ^Ac^isoD7-Аβ_1–16_ (**C**) in the presence of half molar equivalence of Zn^2+^. Peptide concentration is ~0.2 mM. Spectra were recorded at 283K in 90% H_2_O/10% D_2_O in the presence of 10 mM bis-Trid-d_19_ buffer, pH 6.8 at 283K. Characteristic signal at ~0.2 ppm belongs to the methyl group Hγ1* of V12 in dimeric complex. Content of the dimer at ~0.2 mM total concentration of the peptides is about 5%.

**Figure 4:**
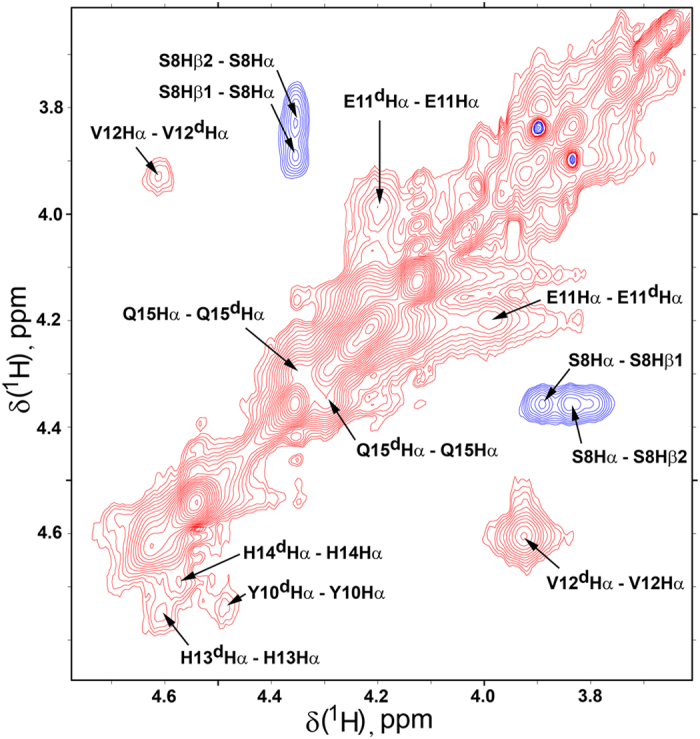
Fragment of the ROESY spectrum (mixing time 100 ms) of the ^Ac^H6R-Аβ_1–16_ in the presence of half molar equivalence of Zn^2+^ recorded at 283 K in D_2_O in the presence of 10 mM bis-Trid-d_19_ buffer, pH 6.8. The peptide concentration ~2.5 mM. Cross-peaks with positive intensity (red) correspond to the exchange signals between monomer and dimer. Negative (blue) cross-peaks correspond to NOE correlations.

Addition of Zn^2+^ ions to all studied peptides leads to substantial line broadening of the NMR signals (Fig. S7). The nature of the peptide signal broadening primarily is associated with the exchange between multiple conformational states of the complex^46^,^47^,^48^. Potentially the aggregation processes can also cause signal broadening. However, the extent of line broadening for peptide H6R-Аβ_1–16_ that never aggregated, and the peptides that aggregate is virtually the same. This means that aggregation does not affect NMR line widths of these peptides under the given conditions. Additionally, an extra set of resonances (Figs 3 and 4, S2 and S3, S8) has been found in NMR spectra of the peptides Аβ_1–16_, H6R-Аβ_1–16_, isoD7-Аβ_1–16_ and Аβ_6–16_ in the presence of zinc ions. This set has been assigned to the dimeric peptide complexes (see below).

### Zinc-induced dimers of Аβ_1–16_, Аβ_6–16_, H6R-Аβ_1–16_ and isoD7-Аβ_1–16_ have common zinc-mediated dimerization interface

We previously showed by a combination of NMR, isothermal titration calorimetry (ITC) and surface plasmon resonance methods that a stable dimeric form of the peptide H6R-Аβ_1–16_ is formed when bound to a zinc ion^36^. Residues E11 and H14 of the two peptide subunits formed the dimerization interface in this complex. For the dimeric form of H6R-Аβ_1–16_ a set of characteristic signals was observed in NMR spectra, including marked resonances of the methyl group Hγ1* of V12 (~0.2 ppm) and amide proton of H14 (~9.5 ppm). The intensity of these signals made it possible to evaluate the dimer ratio, which is dependent on the total peptide concentration and varied between 1–3% for the diluted solutions and 35–40% for the solutions of H6R-Аβ_1–16_ at the peptide concentration of 5–8 mM.

A comparison of the NMR spectra of the peptides Аβ_1–16_, Аβ_6–16_, H6R-Аβ_1–16_ and isoD7-Аβ_1–16_ in the presence of zinc ions obtained in this study has shown a set of characteristic signals similar to that observed in the work cited above, for all these peptides (Fig. 3, S3, S8). At low concentration of the peptides Аβ_1–16_, H6R-Аβ_1–16_ and isoD7-Аβ_1–16_ (~0.2 mM) fraction of the dimeric form is nearly identical (Fig. 3). Fraction of the dimeric forms rises with concentration of the peptides as expected for any dimerization process. Such tendency has been shown for H6R-Аβ_1–16_^36^, which has the highest dimer abundance among the studied peptides. The common set of characteristic signals found for the dimers of Аβ_1–16_, Аβ_6–16_, H6R-Аβ_1–16_ and isoD7-Аβ_1–16_ indicates that all the dimers have the same zinc-induced dimerization interface, namely, _11_EVHH_14_.

### Equilibrium between zinc-bound monomers and dimers of Аβ_1–16_ isoforms

Equilibrium between free peptide and its zinc-bound monomer complex is fast on the NMR time scale. In contrast, equilibrium between zinc-bound monomers and dimers is slow on the NMR time scale, as evidenced by the two separate sets of NMR signals (Fig. 3, 4, S2,S3,S8). The exchange between monomers and dimers is unambiguously confirmed by the rotating frame nuclear Overhauser effect spectroscopy (ROESY) since cross-peaks originating from the through-space dipole-dipole interaction (NOE) have opposite signs to the cross-peaks derived from chemical exchange. Figure 4 illustrates NOEs between S8 Hα and Hβ protons (negative, blue), and exchange cross peaks between several Hα signals of monomeric and dimeric forms (red, positive). Exchange between zinc-bound monomers and dimers of H6R-Аβ_1–16_ and isoD7-Аβ_1–16_ is also confirmed by characteristic cross-peaks in the NOESY spectra (Fig. S8) demonstrating exchange between V12 Hγ1* signals. Both peptides show identical patterns of NMR signals and differ only by fraction of the dimeric forms.

Rate constants *k*_*m*_ _*→*_ _*d*_ and *k*_*d*_ _*→*_ _*m*_ at equilibrium between the monomeric and dimeric complexes of H6R-Аβ_1–16_ with Zn^2+^ have been measured using the magnetization transfer NMR experiments (see Supporting information pp. S24-S28 for details). It has been found that an effective rate constant *k*_*m→d*_ measured at the total peptide concentration of 2.3 mM in the presence of half molar equivalence of ZnCl_2_ equals to 8.6 ± 0.6 s^−1^ and *k*_*d→m*_ equals to 31.3 ± 2.3 s^−1^.

### Structure of zinc-mediated H6R-Аβ_1–16_ dimer in solution

**Figure 5:**
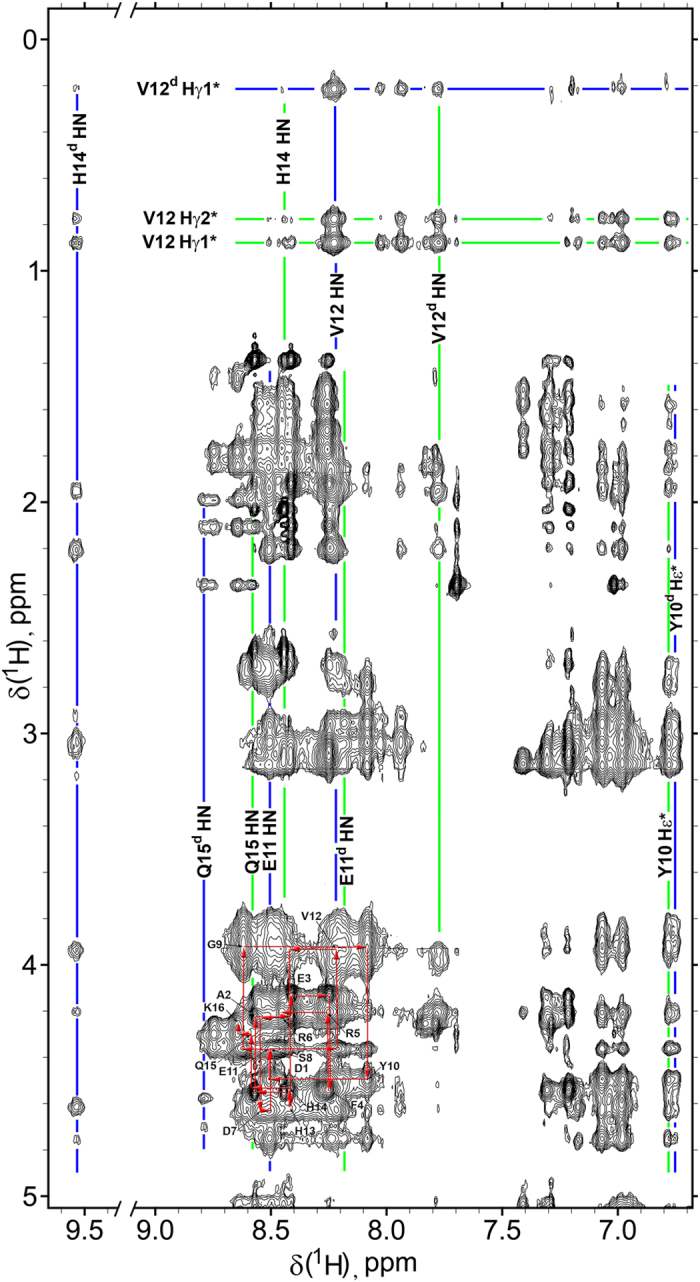
Fragment of the NOESY spectrum (mixing time 250 ms) of the ^Ac^H6R-Аβ_1–16_ in the presence of half molar equivalence of Zn^2+^ recorded at 274 K in 90%H_2_O/10%D_2_O, 10 mM bis-Trid-d_19_ buffer, pH 6.8. The peptide concentration ~2.5 mM. Red arrows show sequential assignment pathway in the region of HN–Hα correlations. Blue lines indicate resonances of the representative signals of the dimer complex, green lines correspond to the signals of the monomeric zinc-peptide complex.

Significant ratio of stable (on NMR time scale) zinc-bound H6R-Аβ_1–16_ dimers allowed to determine the dimer structure in solution. The NMR structure has been determined using the set of distance restraints collected from 2D NOESY and ROESY spectra and a set of constraints between zinc ion and residues E11 and H14 (Table 2). In addition to NOEs between the signals of the dimer, NOEs between the signals of zinc-bound monomer have been also included in the list of distance restraints used for structure calculation (Table 2). Due to the equilibrium between zinc-bound monomer and dimer, more intensive signals of monomer (Fig. 5) contain all the information about interatomic distances within the dimer via the transferred NOE mechanism. Similar transferred NOE mechanism is observed in the equilibrium between free peptide and its complex with a larger protein^49^. Effectiveness of such mechanism is determined by the substantially more effective cross-relaxation in dimer due to its slower molecular tumbling.

QM/MM calculations have been carried out to determine the restraints that describe geometry of the zinc-mediated interface formed by pairs of E11 and H14 from the interacting subunits. An approach similar to that described earlier for the determination of NMR structure of rat Аβ_1–16_ complex with zinc ions^47^ has been used in the calculations. Tetrahedral geometry of the zinc ion coordination sphere originated from quantum mechanics calculations (Fig. S12B). Such geometry is in good agreement with the principles governing Zn binding in proteins^50^ and in agreement with the previously determined structures of Аβ_1–16_ zinc-bound complexes^11^,^17^,^34^,^47^.

Single set of zinc-bound dimer chemical shifts indicates that dimer subunits are chemically equivalent. Therefore, each NOE has been assumed to represent either intra-chain correlation or correlation between two adjacent subunits. All NOEs have been treated in the structure calculations as ambiguous distance restraints allowing optimization protocol to find optimal assignments.

**Figure 6:**
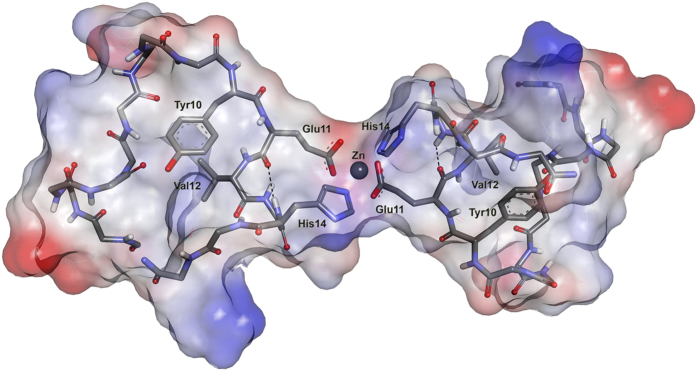
Structure of the representative conformer H6R-Aβ_1–16_ zinc-bound dimer after additional QM/MM refinement. Shown are backbone atoms of two polypeptide chains and side chains of the residues 10–14 involved in formation of zinc-induced dimerization interface. Solvent-accessible molecular surface is calculated for all atoms and colored according to the interpolated charge distribution.

A family of 20 NMR structures has been determined on the basis of 181 experimental restraints (see Table 1 for details) using simulated annealing MD protocol in explicit water environment^51^. For most of the residues, the number of NOEs per residue is between 15 and 40 (Fig. S9). Statistics of the final ensemble are given in Table 1 and superposition of the final family of calculated structures is presented in Fig. S10. A representative structure was selected from the ensemble of calculated structures as being the one that is closest to all the other structures and thus gives the lowest sum of pairwise RMSD for the remainder of the structures in the family. RMSD between the family of calculated structures and the representative structure is about 2.3 Å for the backbone heavy atoms. RMSD between the structures in the final family for heavy atoms of the dimerization interface core (residues 8–15) is about 1.0 Å (Table 1). On Ramachandran plot (Fig. S11), 73.5% of the residues in the whole NMR family are in the most favored regions and none in the disallowed regions. Representative structure has been additionally optimized using the QM/MM method in order to refine the geometry of Zn^2+^ environment (Figs 6 and S12).

### Identification of the second zinc-dependent dimerization interface within Аβ_1–16_, Аβ_6–16_ and isoD7-Аβ_1–16_

Peptides H6R-Аβ_1–16_, Аβ_1–16_, isoD7-Аβ_1–16_ and Аβ_6–16_ have identical primary zinc-mediated homodimerization interface, which shows characteristic resonances, such as Hγ1* of V12 (Figs 3 and S3, S8). In the interface, pairs of residues E11 and H14 of the interacting subunits coordinate a common zinc ion. In the presence of equimolar amounts of zinc ions only H6R-Аβ_1–16_ does not precipitate up to 10 mM, whereas the other peptides rapidly precipitate when their concentration reaches threshold values (>5 mM for Аβ_1–16_, >0.2 mM for isoD7-Аβ_1–16_, >0.8 mM for Аβ_6–16_) (Table 1). Apparently, precipitation can occur only if an oligomer subunit possesses at least two sites that sterically can be involved in the formation of zinc-mediated interfaces with other peptide subunits. Accordingly, the Аβ_1–16_, Аβ_6–16_ and isoD7-Аβ_1–16_ peptides must have a second dimerization interface located within the Аβ minimal zinc binding site 6–14^34^. The fact that aggregation of H6R-Аβ_1–16_ is not observed at any peptide concentration as shown in Table 1, clearly indicates the involvement of residue H6 in the second zinc-dependent interface. Considering that side chains of residues Asp, Glu и His are typical zinc chelators and E11 и H14 are part of the primary dimerization interface, one can rationally assume that the second dimerization interface should include residues D7 (isoD7) or H13 in addition to H6.

To probe involvement of isoD7 in the second dimerization interface we have studied a capacity of the model peptide isoD7-Аβ_1–10_ to form dimers in the presence of zinc ions. We have performed NMR titration experiments using the method of continuous variations^36^,^38^,^52^. The results show that stoichiometry of interaction of isoD7-Аβ_1–10_ with Zn^2+^ is 1:1 (Fig. S13B, Table 1). The coordination centers of zinc ion in the monomeric complex with isoD7-Аβ_1–10_ have been identified from changes of the chemical shifts between free and zinc-bound states (Fig. S14, Tables S5, S6). In addition to imidazole ring of H6, Zn^2+^ is coordinated by the side chain carboxyl group of isoD7 and two backbone carbonyl groups of the residues F4 and H6. Chemical shift changes data (Fig. S15) allowed to calculate the binding constant of Zn^2+^ to isoD7-Аβ_1–10_, K_a_ = 1.19 ± 0.06·10^3^ M^−1^ (Fig. S13A, Table 1). The data show that the peptide forms a monomeric complex with zinc ion and thus isoD7 is not involved in the second dimerization interface.

Similarly, to probe involvement of residue H13 in the second dimerization interface we have studied the model peptide isoD7-H13R-Аβ_1–16_. The H13R substitution has been designed to minimize changes in the peptide properties relative to isoD7-Аβ_1–16_. Chemical shifts of the peptide in the presence and absence of zinc ions are given in Supplementary material (Tables S3 and S4). NMR studies of the interaction of isoD7-H13R-Аβ_1–16_ with Zn^2+^ (Fig. S16) have shown zinc binding constant K_a_ = 1.35 ± 0.06·10^3^ M^−1^ (Fig. S17A, Table 1) and stoichiometry (1:1) (Fig. S17B, Table 1). Zinc-induced chemical shift changes (Fig. S18) indicate that side chains of the two histidine residues (H6 and H14) are involved in the coordination of Zn^2+^. Chemical shifts of E11 do not change upon interaction of the peptide with Zn^2+^, suggesting that this residue is not involved in its coordination. RMSD of the ^1^H and ^13^C chemical shifts between the free and zinc-bound peptides (Fig. S18) indicate that zinc ion is coordinated by backbone carbonyl groups of the residues H6 and R5, similarly to that observed earlier in the S8 phophorylated Aβ_1–16_ peptide^38^.

Taken together, the described data show that in zinc-induced oligomers of the Аβ_1–16_, Аβ_6–16_ and isoD7-Аβ_1–16_ peptides each subunit interacts with the adjacent subunits via residues E11 and H14 from the primary dimerization interface, and residues H6 and H13 from the second one.

### The origins of higher oligomerization propensity of isoD7-Аβ_1–16_ and Аβ_6–16_ compared to Аβ_1–16_

Our experimental results indicate that isomerization of D7 as well as truncation of the first five residues facilitate formation of the second Zn-dependent interaction site. To understand why a relatively small change of the peptide structure between Аβ_1–16_ and isoD7-Аβ_1–16_ causes dramatic change in their zinc-induced oligomerization, MD simulations have been performed for homodimer Aβ_1–16_·Aβ_1–16_ and heterodimer Aβ_1–16_·isoD7-Aβ_1–16_. Conformational behavior of Aβ_1–16_ and isoD7-Aβ_1–16_ chains in the respective homodimer and heterodimer, has been examined in MD trajectories. In the MD simulations the primary zinc-dependent interface structure has been kept in its initial conformation, and distances between the imidazole rings of residues H6 and H13 in the second interface in both Aβ_1–16_ and isoD7-Aβ_1–16_ chains have been constrained in order to keep them close enough for zinc ion coordination.

**Figure 7:**
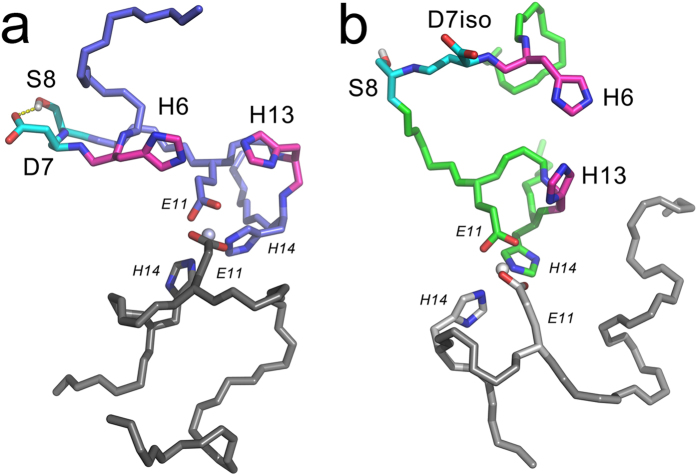
Final conformations from the 20 ns restrained molecular dynamic trajectories of the dimers (**A**) Aβ_1–16_·Aβ_1–16_ and (**B**) isoD7-Aβ_1–16_·Aβ_1–16_. Restraint of 6 Å has been applied to the distance between H6 Nε2 and H13 Nε2 atoms during MD simulations in order to keep positions of imidazole rings in conformation favorable for zinc binding. Residues H6, D7, isoD7, S8 and H13 are shown by bold sticks.

MD simulations have shown that residues D7 and S8 in the Aβ_1–16_ chain form a stable bend (RMSD over N, C and Cα atoms of residues 7 and 8 was 0.13 Å). Such bend is stabilized by hydrogen bond between the D7 side-chain carboxyl and S8 side-chain hydroxyl (Fig. 7a). In contrast, in the isoD7-Aβ_1–16_ chain residues D7 and S8 do not form a bend, but demonstrate a propensity to form extended conformation (Fig. 7b). Thus, structural changes associated with the isomerization of D7 (elongation of the peptide backbone by one additional CH_2_ group and reduction of the side-chain) lead to disruption of the interaction between residues D7 and S8.

MD results show that in both peptides Аβ_1–16_ and isoD7-Аβ_1–16_, residues H6 and H13 adopt a conformation supporting interaction with Zn^2+^. However, probability to adopt the most favorable conformation is higher for the isoD7-Аβ_1–16_ peptide. On the contrary, such conformational space in Аβ_1–16_ peptide is restricted due to the bent region 7–8 (Fig. S19). Thus, isomerization of D7 increases capacity of the residues H6 and H13 to form the second zinc-dependent interface interaction with zinc ion, which facilitates oligomerization of the peptide. Truncation of the first five residues of Аβ_1–16_ forms the Аβ_6–16_ peptide with increased conformational freedom of the N-terminal residue H6 and similarly facilitates zinc-induced oligomerization.

## Discussion

In human tissues, Aβ is a group of peptides, heterogenous at their N-termini and/or C-termini and produced by the β- and γ-secretase-dependent cleavage of amyloid precursor protein^53^. Structural characterization of the full lenght Aβ monomer in solution is difficult due to the tendency of the peptide to aggregate. It was found that the soluble Aβ_1–42_ peptide is an intrinsically disordered polypeptide in aqueous solution, having β-strand secondary structure within the segments 2–7, 16–23, 28–32, and 34–36^54^. Solid-state NMR spectroscopy and electron microscopy allowed to identify diverse morphologies and structures in Aβ fibrils (reviewed in^55^,^56^). The metal-binding domain 1–16 was found to be flexible, situated outside of the hydrophobic core formed by the residues 17–42^20^,^57^. It is worth noting that the absence of this domain in the so called p3 peptide, which comprises the Aβ region 17–42, prevents the formation of oligomers^57^,^58^. Another fact that three amino acid substitutions that discern Aβ of rats and mice from all other mammals and protect rodents against AD are situated within the metal binding domain, indicating its involvement in initialization of the pathological Aβ aggregation. Several AD-causing mutations are also located in the metal binding domain of Aβ^59^,^60^. A role of Aβ_1–16_ in the AD pathology is that it is required for the formation of zinc-induced oligomers. Antibodies targeting the N-terminal region of Aβ are able to block the formation of Aβ40 amyloids^61^, thus suggesting that the N-terminal domain contributes to the formation and/or the maturation of Aβ fibrils.

Our results indicate that conformation of the 10–15 region of the metal binding domain in absence of zinc ions is identical in all studied variants of Aβ_1–16_, and that it is likely to be pre-formed for an effective zinc ion trapping. The region 11–14 was shown to play a principle role in Zn^2+^ binding in the monomeric complex^17^,^34^. Such interaction includes two consecutive steps, (i) primary zinc ion binding by side chains of the residues E11, H13, H14 and a water molecule, (ii) substitution of water by the residue H6 due to movement of the fragments 1–8 and 11–14 towards each other.

It was suggested that a zinc ion binds to monomeric Aβ and then the zinc-peptide complex undergoes conformational changes that lead to the formation in the formation of zinc-induced Aβ oligomers^11^,^21^,^62^,^63^. The role of _11_EVHH_14_ fragment in the formation of Aβ dimers was reported previously^11^,^36^,^37^,^38^,^64^. In the current study we have established the three-dimensional structure of a zinc-linked dimer of H6R-Аβ_1–16_, with the dimerization interface formed by the side chains of residues E11 and H14. Residues 10–15 also contribute to structure stabilization by hydrophobic interactions between Y10 and V12, and by forming hydrogen bonds between the HN protons of H14 and Gln15 and the backbone carbonyl groups of E11 and V12 respectively (Fig. 6, Fig. S12). Low field shifts of these two amide protons are in agreement with this conclusion. Characteristic high field shift of the resonance of Hγ1*of V12 originates from its proximity to the aromatic ring of Y10 in the hydrophobic core and additionally validates the structure. Thus, binding of a zinc ion to the _11_EVHH_14_ fragment leads to formation of the peptide dimer where one zinc ion is coordinated by the residues E11 and H14 from the two interacting peptide chains.

Miller *et al.*^62^ discuss two possible scenarios of the zinc-induced amyloidogenesis. In the first one, metal ions bind amyloid monomers and induce their assembly into oligomers via interactions of the zinc-bound complexes. Alternative mechanism presumes that metal ions bind to pre-formed apo-oligomers of Aβ. Lim *et al.*^13^ showed that zinc binding to Aβ initiates a cascade of conformational changes leading to intermolecular interactions of Aβ via residues 12–21. After an initial structure rearrangement caused by zinc binding, the C-terminal residues readjust their conformation to support effective intermolecular interaction^18^,^21^,^47^,^57^,^58^,^59^,^61^,^65^. Similar structural changes of the metal-free Aβ_1–40_ peptides were also observed in the presence of the preformed oligomers^13^, suggesting that such conformational transitions may constitute a general molecular mechanism of the Aβ amyloidogenesis.

**Figure 8:**
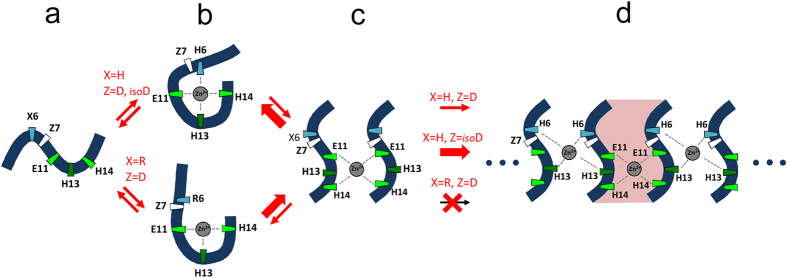
Cartoon representation of the zinc-induced oligomerization of the Aβ metal binding domain. Tubes represent Aβ_1–16_, isoD7-Aβ_1–16_ and Aβ_6–16_ fragments. Zinc ions are shown as gray circles. Shown are stages of oligomerization: (**a**) peptide in free state, (**b**) peptide in complex with zinc ion, (**c**) zinc-induced peptide dimer, and (**d**) zinc-induced peptide oligomer formed around the dimer. Oligomerization seed (peptide dimer) is highlighted by pink.

We have shown here for Aβ_1–16_ and isoD7-Aβ_1–16_ that formation of the primary zinc-dependent dimerization interface by residues E11 and H14 initiates conformational rearrangement leading to formation of the second dimerization interface by residues H6 and H13 (Fig. 7). Emergence of this second interface is a key event enabling zinc-induced oligomerization of the metal binding domain (Fig. 8).

If a zinc-induced dimer is formed by native Aβ_1–16_ via primary interface, it restricts conformational mobility of the peptide. We hypothesize that this can result in disruption of the reciprocal aligning of residues 6 and 13 necessary to form the second interface, leading to lower fraction of peptides with both interfaces (Fig. S19). This explains moderate susceptibility of the native peptide Aβ_1–16_ to oligomerization (Aβ_1–16_ precipitates at concentrations >5 mM, Table 1). In comparison with Aβ_1–16_, isoD7-Aβ_1–16_ undergoes oligomerization at substantially higher rate (isoD7-Aβ_1–16_ precipitates at concentrations >0.3 mM). Isomerization of D7 considerably increases conformational space of the peptide backbone and facilitates favorable aligning of the side chains of residues H6 and H13 that form the second dimerization interface. Another way to increase conformational freedom of the H6 side chain is to remove the first five residues of Aβ, including residues E1 and D3 capable of electrostatically interacting with H6^34^. Indeed, peptide Aβ_6–16_ precipitates at ~0.8 mM (Table 1). Notably, enzymatic removal of the first five Aβ residues is performed by ACE^66^ as we showed earlier, which can potentially link ACE to Alzheimer’s disease.

The results obtained in this study allow to propose the molecular mechanism of zinc-dependent oligomerization of Aβ metal binding domain (Fig. 8). Oligomerization starts with formation of zinc-peptide monomeric complex (Fig. 8b), and subsequent transformation of the complex to a dimer (Fig. 8c), where zinc ion is coordinated by the side chains of residues E11 and H14 from the interacting peptide molecules. After this, conformational rearrangement in the segments 6–14 of each subunit leads to formation of the second zinc-dependent dimerization interface composed of residues H6 and H13. The dimer becomes a seed of subsequent zinc-dependent oligomerization leading to formation of higher order soluble oligomers that are transformed into insoluble aggregates (Fig. 8d). In contrast with the earlier concepts of polymorphism of Aβ within zinc-dependent oligomers, our data suggest that the Aβ metal binding domain has a distinct three-dimensional structure, allowing the domain to simultaneously interact with two other Aβ molecules. This could trigger a “chain reaction” of zinc-induced Aβ oligomerization. This mechanism is in line with our recent *in vivo* studies showing that synthetic peptides specifically binding the Aβ _11_EVHH_14_ region and preventing zinc-induced Aβ dimerization, significantly reduce progression of cerebral amyloidosis in transgenic mice^67^.

## Conclusions

We have demonstrated that in the Aβ metal binding domains of intact, H6R mutant and the isoD7 Aβ isoforms in the absence and presence of zinc ions, the dominant backbone conformation of the fragment 10–15 is identical. This fragment forms primary zinc-mediated dimerization interface of all studied metal binding domains. Solution structure of zinc-mediated H6R-Аβ_1–16_ dimer has been determined, providing insight into the mechanism of formation of the dimerization interface. Zinc-induced oligomerization of synthetic peptides representing the 1–16 metal binding domains of natural Аβ variants has been shown to follow the same molecular mechanism: (i) peptide dimer is formed through the primary zinc-mediated interface _11_EVHH_14_; (ii) residues H6 and H13 are realigned creating the second zinc-dependent interface in each subunit; (iii) the dimer becomes a seed of subsequent zinc-dependent oligomerization of Aβ_1–16_. Our results indicate that the extent of conformational freedom of residue H6 determines the propensity of Aβ_1–16_ isoforms to undergo zinc-induced oligomerization. Targeting of Aβ zinc-mediated interfaces may provide a therapeutic route for AD treatment.

## Methods

### Materials

All chemicals and solvents were of HPLC-grade or better and were obtained from Sigma-Aldrich (St. Louis, MO, USA). Synthetic peptides (purity > 98% checked by RP-HPLC) Аβ_1–16_ (DAEFRHDSGYEVHHQK), Аβ_6–16_ (HDSGYEVHHQK), H6R-Аβ_1–16_ (DAEFRRDSGYEVHHQK), isoD7-Аβ_1–16_ (DAEFRH[isoD]SGYEVHHQK, where [isoD] - isoaspartate), isoD7-H13R-Аβ_1–16_ (DAEFRH[isoD]SGYEVRHQK, and isoD7-Аβ_1–10_ (DAEFRH[isoD]SGY) were purchased from Biopeptide Co., LLC (San Diego, CA, USA). C-termini of all the peptides were protected with amide group. N-termini of Аβ_1–16_ and H6R-Аβ_1–16_ were either unprotected or protected with acetyl. N-terminus of Аβ_6–16_, isoD7-Аβ_1–16_, isoD7-H13R-Аβ_1–16_ and isoD7-Аβ_1–10_ were protected with acetyl group. The lyophilized peptides were dissolved in buffer before each experiment. Concentration of the peptides was determined by UV absorption spectroscopy using spectrophotometer Cary50 (Varian) and the extinction coefficient of 1280 M^−1^ cm^−1^ at 280 nm (from Tyr 10 of Aβ). Zinc chloride (99.99%, ACROS Organics) prior to the weighing was dried out during 1–2 hours at 150 °C.

### Dynamic light scattering

Dynamic light scattering (DLS) measurements were carried out on a Zetasizer Nano ZS apparatus (Malvern Instruments Ltd., UK) in accordance with the manufacturer instruction. The 120-μL aliquots of peptide solutions were placed into a BRAND UV microcuvetter (BRAND GMBH, Germany) and used for the measurements. Measurements of peptides in the presence of Zn^2+^ were carried out within 10 minutes after addition of two-fold molar excess of ZnCl_2_ to the peptide solutions. The instrument is equipped with a He-Ne laser source (λ=632.8 nm) and operates in the back-scatting mode, measuring particle size in the range between 0.6 nm and 10 μm. Particle size distribution was estimated using a CONTIN data analysis utility with spherical approximation of the particles, available as a part of the instrument software.

### Turbidity measurements

Measurements were performed on a NanoDrop 1000 spectrophotometer (Thermo Scientific, USA). The optical density of peptide solutions was measured at 350 nm and 405 nm, using 2 μL aliquots of the peptide solutions. Measurements of peptides in the presence of Zn^2+^ were carried out after 30–40 minutes following addition of ZnCl_2_ to the peptide solutions.s.

### Isothermal titration calorimetry (ITC)

The thermodynamic parameters of zinc binding to Aβ_6–16_ were measured using a MicroCal iTC200 System (GE Healthcare Life Sciences, USA) as described previously^36^,^43^,^47^. Experiments were carried out at 25 °C in 50 mM Tris buffer, pH 7.3. 2 μL aliquots of the ZnCl_2_ solution were injected into the 0.2 mL cell containing the peptide solution to obtain a complete binding isotherm. The titration curves were fitted using MicroCal Origin software. Association constant (K_a_), binding stoichiometry and enthalpy were determined by a non-linear regression fitting procedure (Fig. S20).

### NMR experiments

Peptides in the concentration of 0.2–2 mM were dissolved in 10–20 mM bis-Tris-d_19_ (2,2-Bis(hydroxymethyl)-2,2′,2″-nitrilotriethanol-d_19_ with 98% ^2^D enrichment) buffer solution (pH 6.8). Sodium salt of 3-(trimethylsilyl)propionic-2,2,3,3-d_4_ acid in the concentration of 10–40 μM was added as a standard. NMR spectra were measured in the temperature range between 274 K and 308 K either in D_2_O or in 90% H_2_O/10% D_2_O on a Bruker AVANCE 600 MHz spectrometer equipped with a triple resonance (^1^H, ^13^C and ^15^N) pulsed field z gradient probe or on a Varian INOVA 600 MHz spectrometer equipped with a triple resonance (^1^H, ^13^C and ^15^N) cryoprobe. 1D NMR spectra were processed and analyzed using the Mnova software (Mestrelab Research, Spain). 2D NMR spectra were processed by NMRPipe^68^ and analyzed using SPARKY^69^.

### NMR signal assignment

The ^1^H, ^15^N and ^13^C signal assignments of isoD7-Аβ_1–10_, isoD7-Аβ_1–16_, isoD7-H13R-Аβ_1–16_ and Аβ_6–16_ peptides in free form and in complex with zinc ions were obtained using the following 2D spectra: DQF-COSY, TOCSY (mixing time of 70 and 80 ms), NOESY (mixing time of 200, 225, 250 and 300 ms), ^13^C-^1^H HSQC and ^15^N-^1^H HSQC. Heteronuclear experiments were measured at the natural abundance of the ^13^C and ^15^N isotopes (Fig. S1–S4). Chemical shifts are presented in the supporting material (Tables S1–S7).

### NMR titration experiments

To identify the amino acid residues that coordinate zinc ion in the peptides isoD7-Аβ_1–10_ and isoD7-H13R-Аβ_1–16_, and for determining association constants of zinc ions NMR titration experiments were carried out. Peptides at concentration of 1.0–1.5 mM at pH 6.8–7.0 were titrated with a solution of ZnCl_2_ in a buffer of identical composition at the same pH value. 1D spectra were recorded for each titration point. Figures S15 and S16 show changes of the chemical shifts with increasing molar content of [Zn^2+^] from 0.05 to 10.0 relatively to the peptide concentration. The volume of solution at the final point increased from 600 to 800 μL. Chemical shift changes of the representative signals in titration experiments were used for calculation of the K_a_ values (Figs S13A and S17A). The linear nature of Δδ values Δδ presented in Scatchard coordinates^52^ confirms equilibrium between the free and zinc-bound peptide forms.

Method of continuous variations^52^ was used to determine stoichiometry of zinc binding to the peptides isoD7-Аβ_1–10_ and isoD7-H13R-Аβ_1–16_. This involved preparation of a series of samples containing both the peptide and ZnCl_2_ in varying proportions of the components, but in a fixed total concentration (from 1.0 to 1.7 mM). Changes of the chemical shifts induced by the interaction of the peptide with zinc ions were analyzed. The plots of the product Δδ·[P]_0_ (Δδ – change of the chemical shift; [P]_0_ – total peptide concentration in the sample) versus the mole fraction of [Zn^2+^] show maximum at the fraction value, which corresponds to the stoichiometry (Figs S13B and S17B).

### Magnetization transfer experiments

In order to measure exchange rates between monomeric and dimeric states of the Zn·H6R-Аβ_1–16_ complex, magnetization transfer NMR technique was used. Magnetization transfer experiments involved selective excitation of the signal at 0.9 ppm, which belongs to the methyl group Hγ1* of V12 in the monomeric form of zinc-peptide complex, with subsequent detection of the intensity of the signal at 0.2 ppm, which belongs to the same group in the dimeric form. Series of experiments with varying delays between the inverting and reading pulses were carried out. All the experimental details and data analysis are provided in the supplementary material (pages S24-S28).

### NMR restrains

NOE distance restraints used in structure calculation of the dimeric form of Zn-H6R-Аβ_1–16_ complex were obtained from 2D NOESY spectra acquired at 274 K in H_2_O or at 278 K in D_2_O. NMR signal assignment of Zn···H6R-Аβ_1–16_ complex was reported earlier^36^. Representative fragment of the NOESY spectrum with some principal assignments is shown on Fig. 5. Signal intensities in NOESY spectra were calibrated and converted into the distance restrains using the fixed distance intraresidue NOEs as the reference. Distance and torsion angle restraints representing the coordination site of zinc ion were obtained using the quantum mechanical calculations (see below).

### QM/MM and restrained molecular dynamics

Structures of the complex of H6R-Аβ_1–16_ with zinc ions were determined using the GROMACS 3.3.1 software^70^, AMBER 03 force field^71^ and optimized protocol of the simulated annealing MD calculations^47^,^51^ with the set of distance restraints listed in Table 2. QM/MM Car–Parrinello simulation approach^72^,^73^ was applied to optimize the zinc binding site and to obtain restrains that describe geometry of Zn^2+^ coordination center. All the parameters and protocols of the MD and QM/MM calculations were described earlier in detail^47^. Convergence of the calculations was determined using root mean square deviation (RMSD) of the coordinates of the C, Cα, and N atoms of protein backbone, calculated for the whole family of structures. Quality of the calculated structures was defined on the basis of the percent of hits of the main dihedral angles φ and ψ in the most favorable and prohibited areas of Ramachandran map using Procheck_NMR^74^. Structures were visualized and analyzed using the InsightII or Discovery Studio software (Accelrys Inc.).

### MD simulations

Molecular modeling has been performed for homodimer Aβ_1–16_·Aβ_1–16_ and heterodimer Aβ_1–16_·isoD7-Aβ_1–16_ using the representative NMR conformer of the H6R-Aβ_1–16_ dimer as initial structure. Homology modeling was performed using the Chimera software^75^. Partial charges for the non-standard residue isoD7 were assigned using the AM1-BCC method^76^. Initial models were refined using the restrained molecular dynamic simulation with GROMACS 4.6.5 software^70^ and Amber99SB-ILDN force field^77^. Peptide dimers were placed in the cubic cell with a minimum distance (0.8 nm) between a protein and the box wall and soaked with TIP3P water molecules^78^. Total charge of the solution has been neutralized with sodium ions. Energy was minimized using the steepest descent algorithm and the system was further equilibrated during 100 ps of constant volume (NVT) molecular dynamic simulation followed by 100 ps of constant pressure (NPT) molecular dynamics. 10 ns MD simulations were carried out using the NPT ensemble to relax protein chains. 6 Å restraint has been applied to the distance between H6 Nε2 and H13 Nε2 atoms. 20 ns NPT MD simulations were performed to follow approaching of imidazole rings of H6 and H13. Position of the side chains of residues E11 and H14 together with the coordinated zinc ion were restrained during all simulation steps. Calculations were performed at 300 K, pressure 1 bar, with a 2 fs integration step using Berendsen barostat and velocity rescale method for thermostat. Particle-mesh Ewald method^79^ has been implemented to treat long-range electrostatic interactions, and LINCS algorithm to control the lengths of covalent bonds^80^.

## Additional Information

**Accession Code**: The structural data and experimental restraints used in the calculations have been submitted to the Protein Data Bank with accession number 2MGT.

**How to cite this article**: Istrate, A. N. *et al.* Interplay of histidine residues of the Alzheimer's disease Aβ peptide governs its Zn-induced oligomerization. *Sci. Rep.* **6**, 21734; doi: 10.1038/srep21734 (2016).

## Supplementary Material

Supplementary Information

## Figures and Tables

**Figure 1:**
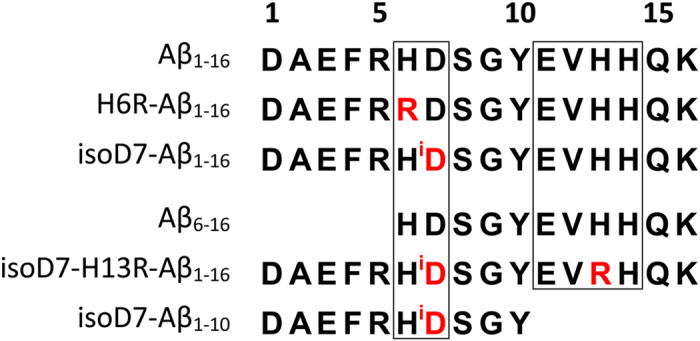
Amino acid sequences of the studied Аβ fragments.

**Table 1:** Parameters of zinc binding to the peptides measured by ITC or NMR, mean diameter of oligomers in the absence and presence of twofold molar excess of ZnCl_2_ measured by dynamic light scattering, and turbidity of the solutions.

Peptides	Zinc binding			C, mM	DLS	Turbidity^c^ OD_350_ × 10^2^/OD_405_ × 10^2^	
	K_a_ × 10^4^, M^−1^	N^a^	Ref		Diameter, nm^b^	Free peptide	Peptide + ZnCl_2_
Aβ_1–16_	1.70 ± 0.40	1.0	ITC 43	0.2	0	2.2 ± 1.3/2.2 ± 1.8	3.0 ± 1.7/1.8 ± 0.9
				5.0	2.5 ± 0.3	9.4 ± 1.7/5.4 ± 1.1	44 ± 10/38 ± 10
H6R-Aβ_1–16_	0.24 ± 0.03	0.5	ITC^36^	1.0	0	1.5 ± 0.6/1.7 ± 0.9	2.0 ± 1.2/1.9 ± 1.3
isoD7-Aβ_1–16_	1.30 ± 0.40	1.0	ITC 43	0.8	2.7 ± 0.3	2.4 ± 1.1/2.4 ± 1.1	491 ± 232/454 ± 219
Aβ_6–16_	1.27 ± 0.13	1.0	this work, ITC	2.0	17 ± 6	2.0 ± 0.8/2.2 ± 0.8	422 ± 20/278 ± 11
isoD7-H13R-Aβ_1–16_	0.14 ± 0.01	1.0	this work, NMR	1.0	0	1.7 ± 1.1/1.4 ± 1.0	2.0 ± 1.3/2.0 ± 1.1
isoD7-Aβ_1–10_	0.12 ± 0.01	1.0	this work, NMR	1.0	0	2.6 ± 1.4/2.5 ± 1.1	2.8 ± 1.7/2.8 ± 1.3

Concentration of the peptides in DLS and turbidity measurements are shown.

^a^N – stoichiometry.

^b^Mean diameter of oligomers determined by DLS.

^c^OD_350_ and OD_405_ – optical density of the peptide solutions measured at 350 nm and 405 nm.

**Table 2:** NMR restrains and structural statistics for the complex of two H6R-Aβ_1–16_ peptides with zinc ion.

Number of NOE restrains	181
Intra-residue	91
Sequential	55
Medium-range	23
Long-range (|i-j| > 4)	4
Distance constraints of Zn^2+^ chelating center	8
Ramachandran map statistics	
% of residues in most favorable region of Ramachandran map	73.5
% of residues in disallowed region of Ramachandran map	0.0
NMR family statistics	
Number of conformers in family of structure	20
Number of distance violations (>0.5 Å) per structure	<1
Backbone (C, Cα and N) rmsd of residues 1–16 (Å)	2.31 ± 0.55
Backbone (C, Cα and N) rmsd of residues 6 – 15 (Å)	1.39 ± 0.36
Backbone (C, Cα and N) rmsd of residues 8 – 15 and all heavy atoms of residues 11 and 14 (Å)	1.08 ± 0.26
