# Supplementary material for: Interplay of histidine residues of the Alzheimer’s disease Aβ peptide governs its Zn-induced oligomerization
Source: Sci Rep. 2016 Feb 22;6:21734. doi: 10.1038/srep21734 (PMC4761979; doi:10.1038/srep21734)
Supplement: Supplementary Information [file srep21734-s1.pdf]

Supplementary Information

Interplay of histidine residues of the Alzheimer's disease Aβ peptide governs its Zn-induced oligomerization

Andrey N. Istrate,[1,*] Sergey A. Kozin,[1,*] Sergey S. Zhokhov,[2] Alexey B. Mantsyzov,[2] Olga I. Kechko,[1] Annalisa Pastore,[3] Alexander A. Makarov[1] and Vladimir I. Polshakov[2,†]

[1] *Engelhardt Institute of Molecular Biology, Russian Academy of Sciences, 119991, Moscow, Russia;*

[2] *Faculty of Fundamental Medicine, M.V. Lomonosov Moscow State University, 119991, Moscow, Russia;*

[3] *National Institute for Medical Research, Medical Research Council, The Ridgeway, Mill Hill, NW7 1AA, London, UK.*

* These authors contributed equally to this work

†*E.mail*: vpolsha@mail.ru

Table of contents

		Page
**Figure S1**	2D ^1H-^{15}N HSQC spectrum of ^{Ac}isoD7-Aβ_{1-16} recorded at a natural abundance of the ^{15}N nuclei at 283K.	S4
**Figure S2**	Cα-Hα region of 2D ^1H-^{13}C HSQC spectra of the free peptide ^{Ac}H6R-Aβ_{1-16} and its complex with Zn^{2+} recorded at a natural abundance of the ^{13}C nuclei at 283K.	S5
**Figure S3**	Fragments of 2D ^1H-^{13}C HSQC spectra of the peptides ^{Ac}H6R-Aβ_{1-16} and Aβ_{6-16} in free state and in the presence of twofold molar excess of Zn^{2+}.	S6
**Figure S4**	Overlay of the 2D ^1H-^{15}N HSQC spectra of the free peptide ^{Ac}isoD7-Aβ_{1-10} and its complex with Zn^{2+} recorded at a natural abundance of the ^{15}N nuclei at 283K.	S7

**Figure S5**	Fragments of NOESY spectra (mixing time 250 ms) of the free peptides A$\beta_{1\text{-}16}$, H6R-A$\beta_{1\text{-}16}$, and ^{Ac}isoD7-A$\beta_{1\text{-}16}$ illustrating sequential and medium-range NOEs.	S8
**Figure S6**	Fragments of NOESY spectra (mixing time 250 ms) of the free peptides A$\beta_{1\text{-}16}$, H6R-A$\beta_{1\text{-}16}$, and ^{Ac}isoD7-A$\beta_{1\text{-}16}$ illustrating NOEs between HN signals.	S9
**Figure S7**	Region of amide and aromatic signals of ^{1}H NMR spectra of isoD7-A$\beta_{1\text{-}16}$ in free state and after addition of equimolar amount of $ZnCl_2$.	S10
**Figure S8**	Fragment of NOESY spectra of the ^{Ac}H6R-A$\beta_{1\text{-}16}$and ^{Ac}isoD7-A$\beta_{1\text{-}16}$ in the presence of half molar equivalence of Zn^{2+}.	S11
**Figure S9**	Plot of the number and distribution of NOEs versus the amino acid sequence (one chain) that were used in the structure calculation of ^{Ac}H6R-A$\beta_{1\text{-}16}$.	S12
**Figure S10**	Ensemble of the final 20 calculated NMR structures of the complex of ^{Ac}H6R-A$\beta_{1\text{-}16}$ with Zn^{2+}.	S13
**Figure S11**	Ramachandran plot for the final 20 structures of the complex of ^{Ac}H6R-A$\beta_{1\text{-}16}$ with Zn^{2+}.	S14
**Figure S12**	Representative structure of the complex of ^{Ac}H6R-A$\beta_{1\text{-}16}$ with Zn^{2+}.	S15
**Figure S13**	Interaction of zinc ions with the peptide isoD7-A$\beta_{1\text{-}10}$. Changes of the chemical shifts during titration of the peptide by $ZnCl_2$ shown in Scatchard coordinates, and the results of Zn^{2+} isomolar NMR titration studies.	S16
**Figure S14**	Chemical change RMSD between the free and Zn-bound states of ^{iso}D7-Aβ(1-10).	S17
**Figure S15**	^{1}H spectra illustrating NMR titration of ^{iso}D7-A$\beta_{1\text{-}10}$ by $ZnCl_2$.	S18
**Figure S16**	^{1}H spectra illustrating NMR titration of isoD7,H13R-A$\beta_{1\text{-}10}$.	S19
**Figure S17**	Interaction of zinc ions with the peptide isoD7,H13R-A$\beta_{1\text{-}10}$. Changes of the chemical shifts during titration of the peptide by $ZnCl_2$ shown in Scatchard coordinates, and the results of Zn^{2+} isomolar NMR titration studies.	S20

**Figure S18**	Chemical change RMSD between the free and Zn-bound states of ^{iso}D7, H13R-Aβ(1-16).	S21
**Figure S19**	Snapshots from 20 ns restrained molecular dynamic trajectories taken with 1 ns step for $Aβ_{1-16}$···$Aβ_{1-16}$ and $Aβ_{1-16}$···isoD7-$Aβ_{1-16}$.	S22
**Figure S20**	ITC titration curve and the binding isotherm for the zinc interaction with $Aβ_{6-16}$.	S23
**Methods**	Determination of the exchange rates between monomeric and dimeric complexes of ^{Ac}H6R-$Aβ_{1-16}$.	S24
**Table S1**	Chemical shifts (ppm) of the ^{1}H, ^{13}C and ^{15}N signals of free human peptide isoD7-$Aβ_{1-16}$.	S29
**Table S2**	Chemical shifts (ppm) of the ^{1}H and ^{13}C signals of isoD7-$Aβ_{1-16}$ in the presence of zinc ions.	S30
**Table S3**	Chemical shifts (ppm) of the ^{1}H, ^{13}C and ^{15}N signals of free peptide isoD7-H13R-$Aβ_{1-16}$.	S31
**Table S4**	Chemical shifts (ppm) of the ^{1}H, ^{13}C and ^{15}N signals of isoD7-H13R-$Aβ_{1-16}$ in the presence of zinc ions.	S32
**Table S5**	Chemical shifts (ppm) of the ^{1}H, ^{13}C and ^{15}N signals of free peptide isoD7-$Aβ_{1-10}$.	S33
**Table S6**	Chemical shifts (ppm) of the ^{1}H, ^{13}C and ^{15}N signals of the peptide isoD7-$Aβ_{1-10}$ in the presence of zinc ions.	S34
**Table S7**	Chemical shifts (ppm) of the ^{1}H, ^{13}C and ^{15}N signals of free peptide $Aβ_{6-16}$.	S35

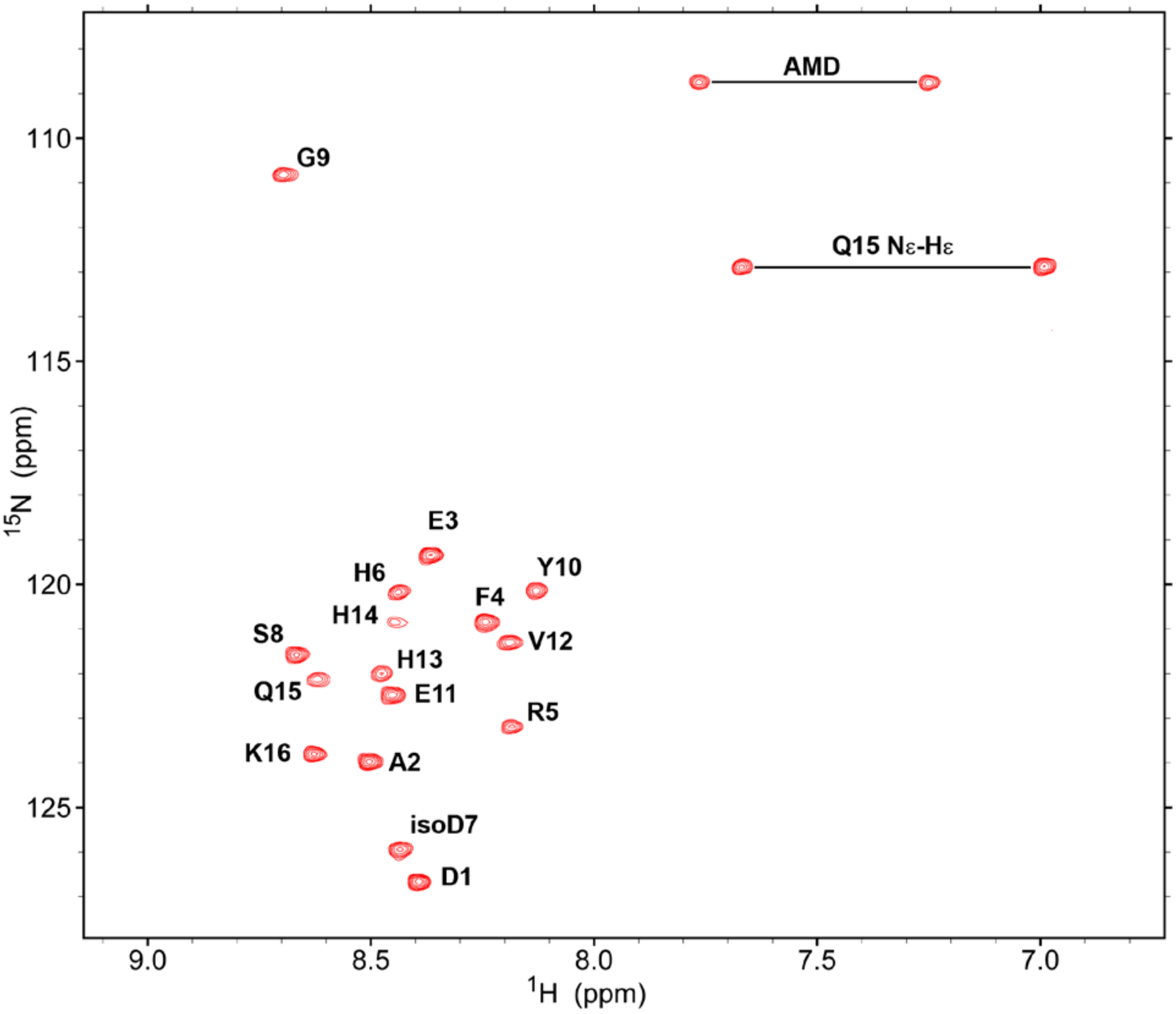

Figure S1. 2D ^{1}H-^{15}N HSQC spectrum of ^{Ac}D7^{iso}-A$\beta_{1\text{-}16}$ recorded at a natural abundance of the ^{15}N nuclei at 283K. The peptide concentration was ~2.2 mM. Spectrum was recorded in H_2O in the presence of 10 mM bis-Trid-d_{19} buffer, pH 6.8.

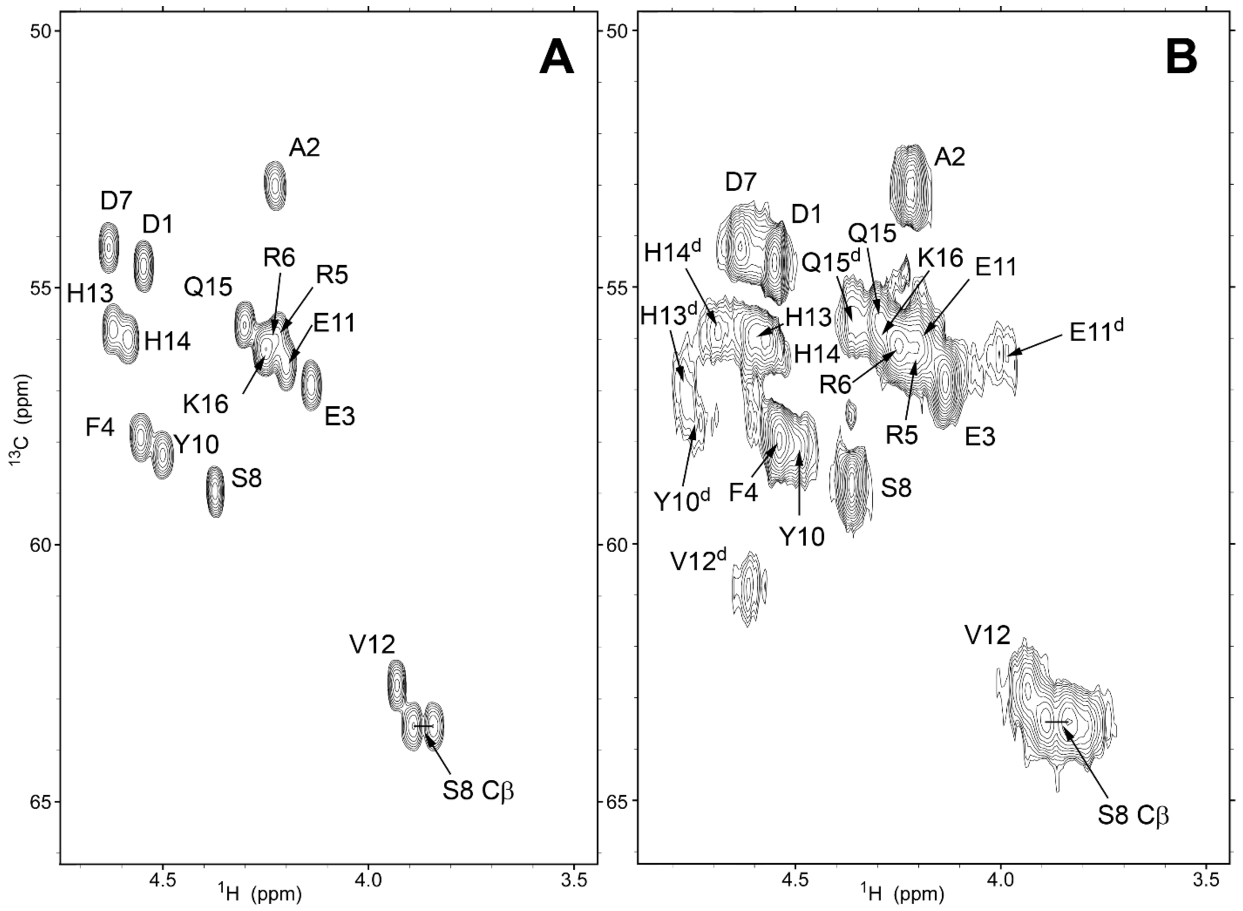

Figure S2. Fragments of 2D ^{1}H-^{13}C HSQC spectra of the free peptide ^{Ac}H6R-A$\beta_{1\text{-}16}$ (A) and its complex with Zn^{2+} (B) recorded at a natural abundance of the ^{13}C nuclei at 283K. Shown are the regions of Cα-Hα correlations. Residues indicated by the symbol d correspond to the dimer resonances. The peptide concentration was ~2.5 mM. Spectra were recorded in D_2O in the presence of 10 mM bis-Trid-d_{19} buffer, pH 6.8.

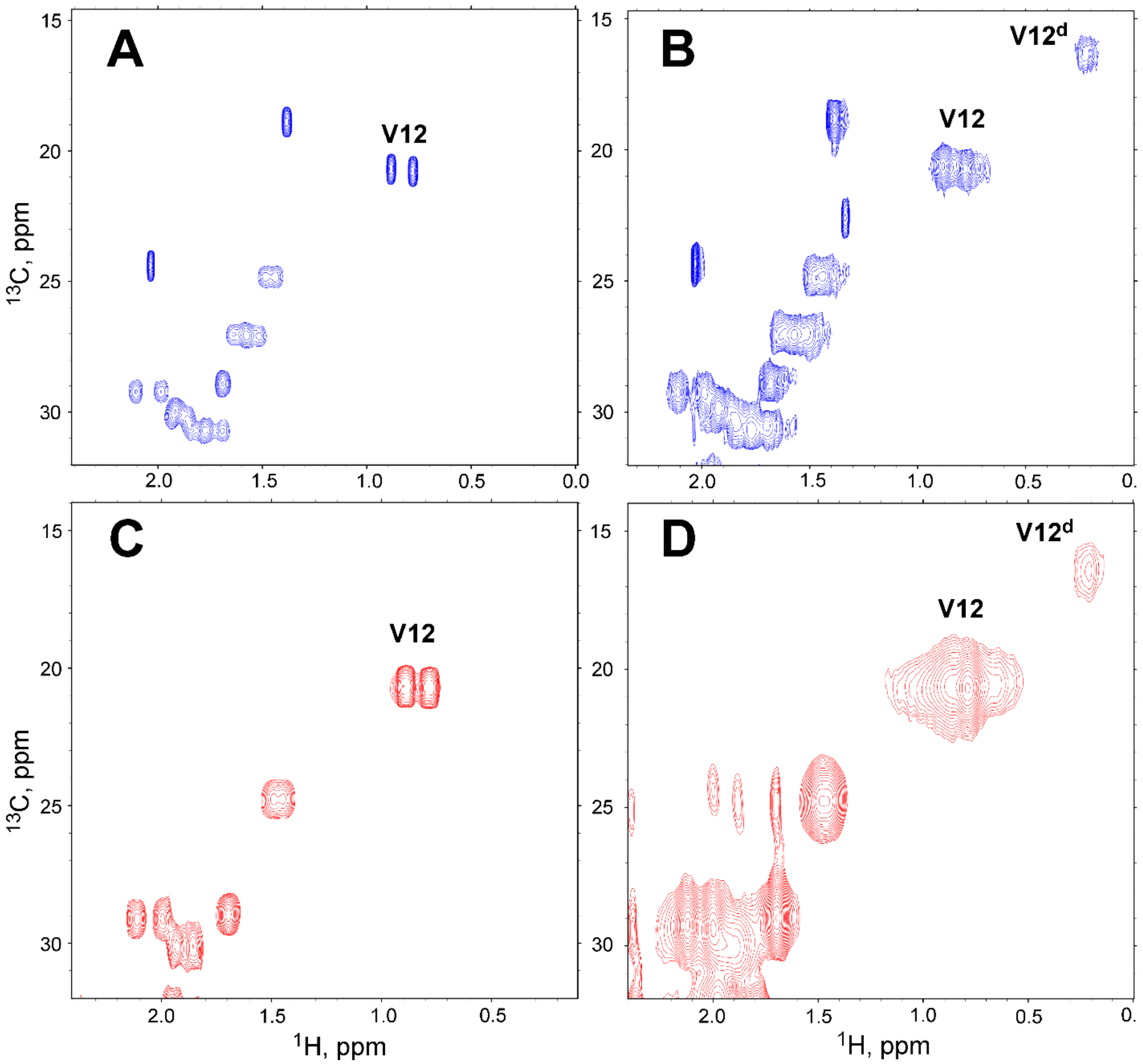

Figure S3. Fragments of 2D ^{1}H-^{13}C HSQC spectra of the free peptide ^{Ac}H6R-Aβ$_{1-16}$ (A), peptide ^{Ac}H6R-Aβ$_{1-16}$ in the presence of twofold molar excess of Zn^{2+} ions (B), free peptide Aβ$_{6-16}$ (C) and peptide Aβ$_{6-16}$ in the presence of twofold molar excess of Zn^{2+}. Spectra were recorded at 283K. Shown are the high-field regions of C-H correlations. Resonances of methyl groups of V12 in free peptides, and monomeric and dimeric zinc-peptide complexes are labeled. The concentration of ^{Ac}H6R-Aβ$_{1-16}$ peptide was ~2.5 mM, concentration of Aβ$_{6-16}$ peptide was ~0.4 mM. Spectra were recorded in the presence of 10 mM bis-Trid-d$_{19}$ buffer, pH 6.8.

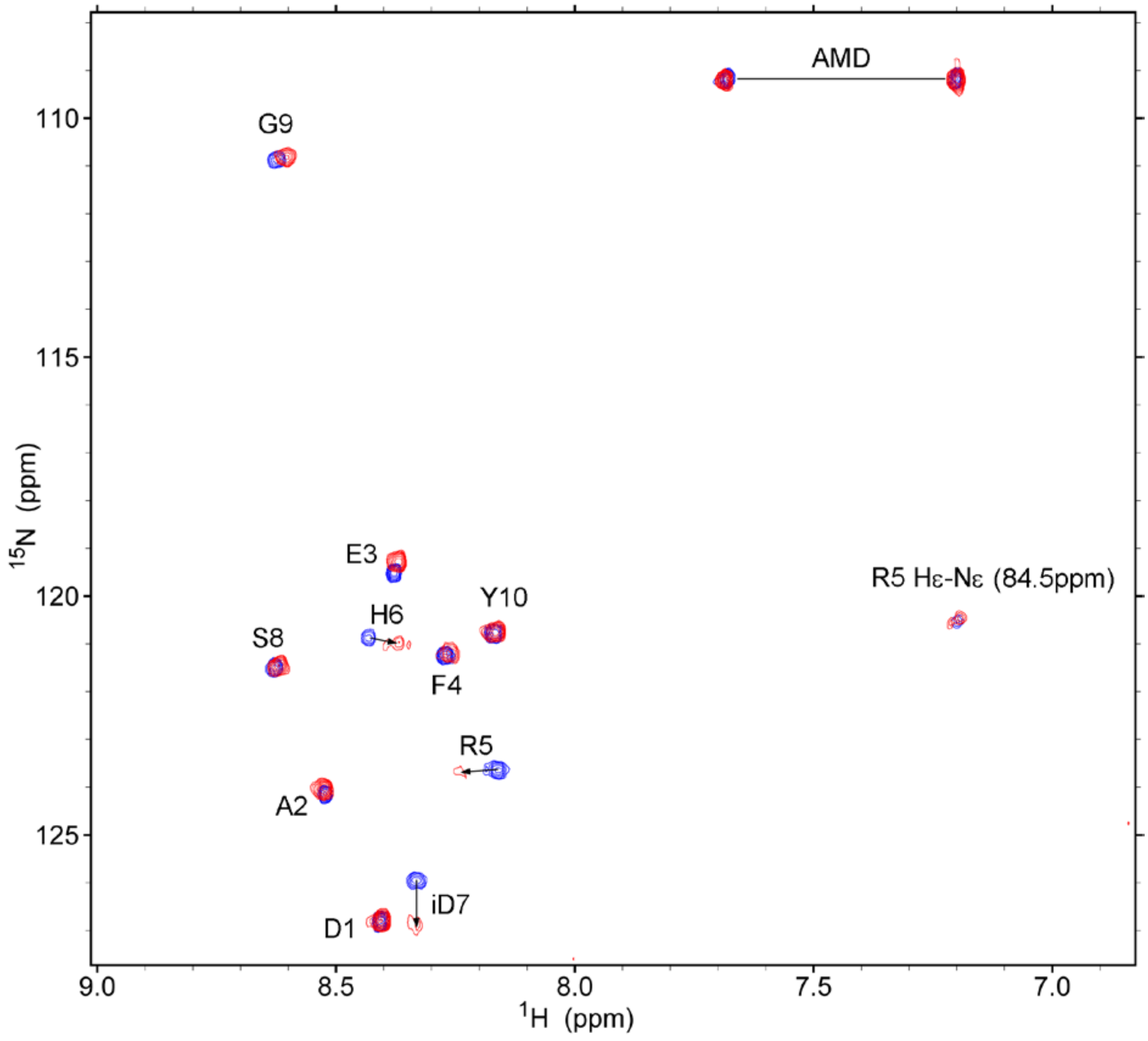

Figure S4. Overlay of the 2D ^{1}H-^{15}N HSQC spectra of the free peptide $^{Ac}D7^{iso}$-A$\beta_{1\text{-}10}$ (blue) and its complex with Zn^{2+} (red) recorded at a natural abundance of the ^{15}N nuclei at 283K. Arrows indicate zinc-induced shifts of the HN resonances. The peptide concentration was ~2.5 mM. Spectra were recorded in 90% H_2O/10% D_2O in the presence of 10 mM bis-Trid-d_{19} buffer, pH 6.8.

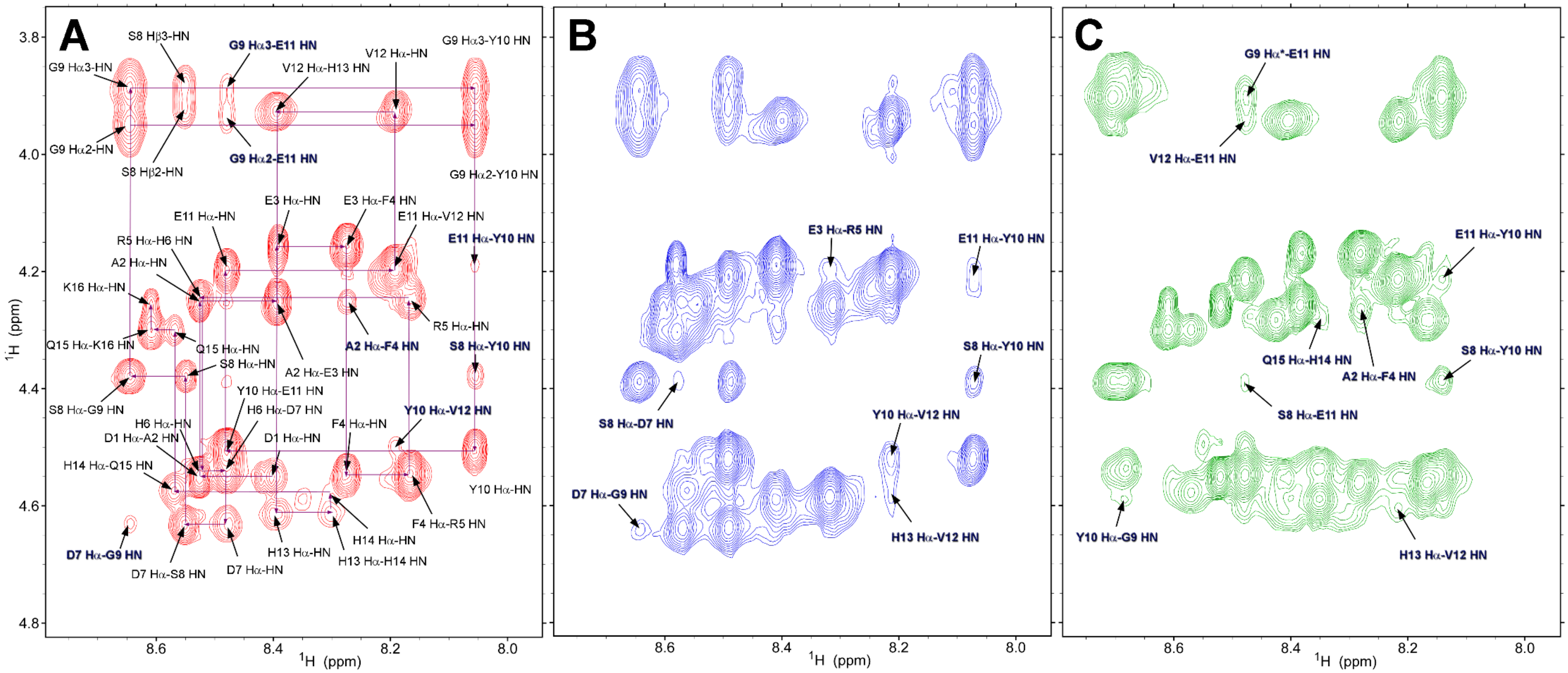

Figure S5. Fragments of NOESY spectra (mixing time 250 ms) of the free peptides Aβ$_{1\text{-}16}$ (A), H6R-Aβ$_{1\text{-}16}$ (B), and ^{Ac}D7^{iso}-Aβ$_{1\text{-}16}$ (C) illustrating sequential and medium-range NOEs, typical for left-handed helix. For the Aβ$_{1\text{-}16}$ (A) purple arrows show pathway of the sequential assignment. NOESY spectra were recorded in 90% H_2O/10% D_2O, in the presence of 10 mM bis-tris-d$_{19}$, pH 6.8, at 283K.

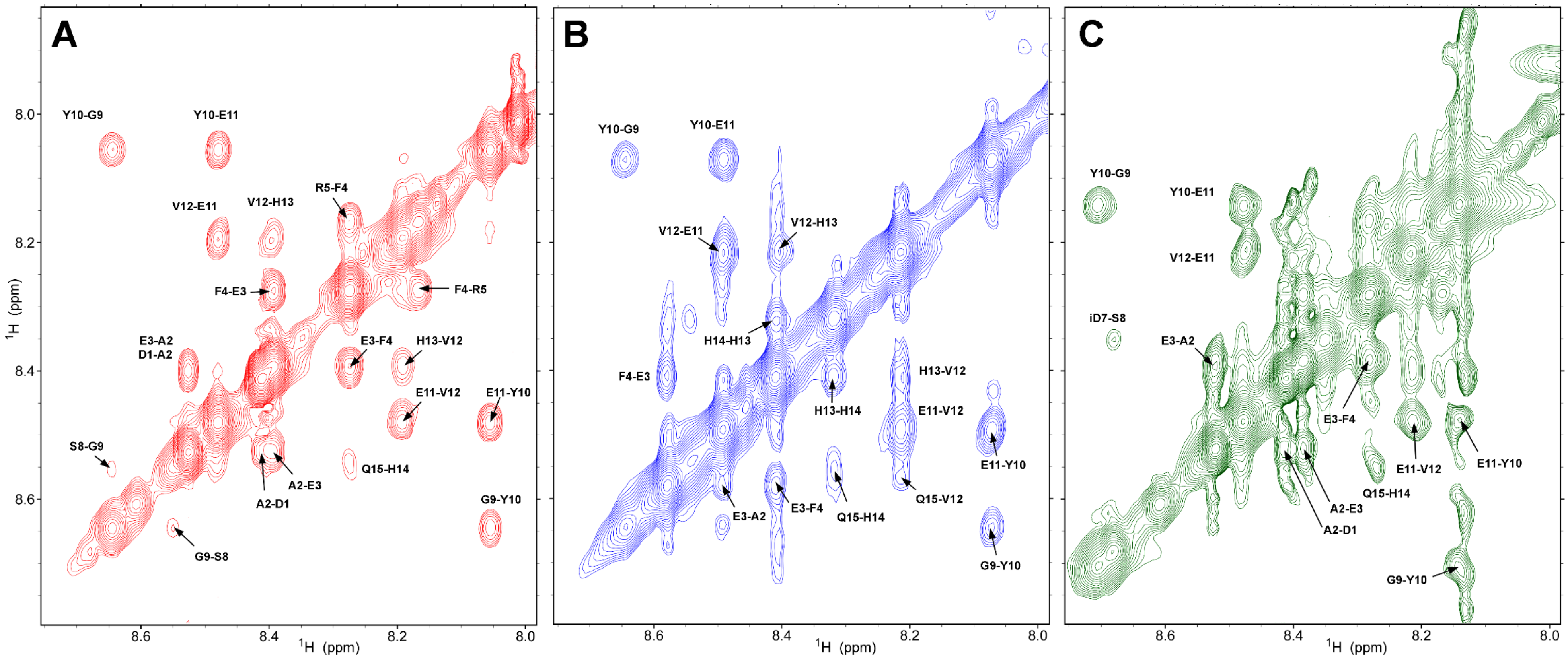

Figure S6. Fragments of NOESY spectra (mixing time 250 ms) of the free peptides Aβ$_{1\text{-}16}$ (A), H6R-Aβ$_{1\text{-}16}$ (B), and ^{Ac}D7^{iso}-Aβ$_{1\text{-}16}$ (C) illustrating NOEs between HN signals. NOESY spectra were recorded in 90% H_2O/10% D_2O, in the presence of 10 mM bis-tris-d$_{19}$, pH 6.8, at 283K.

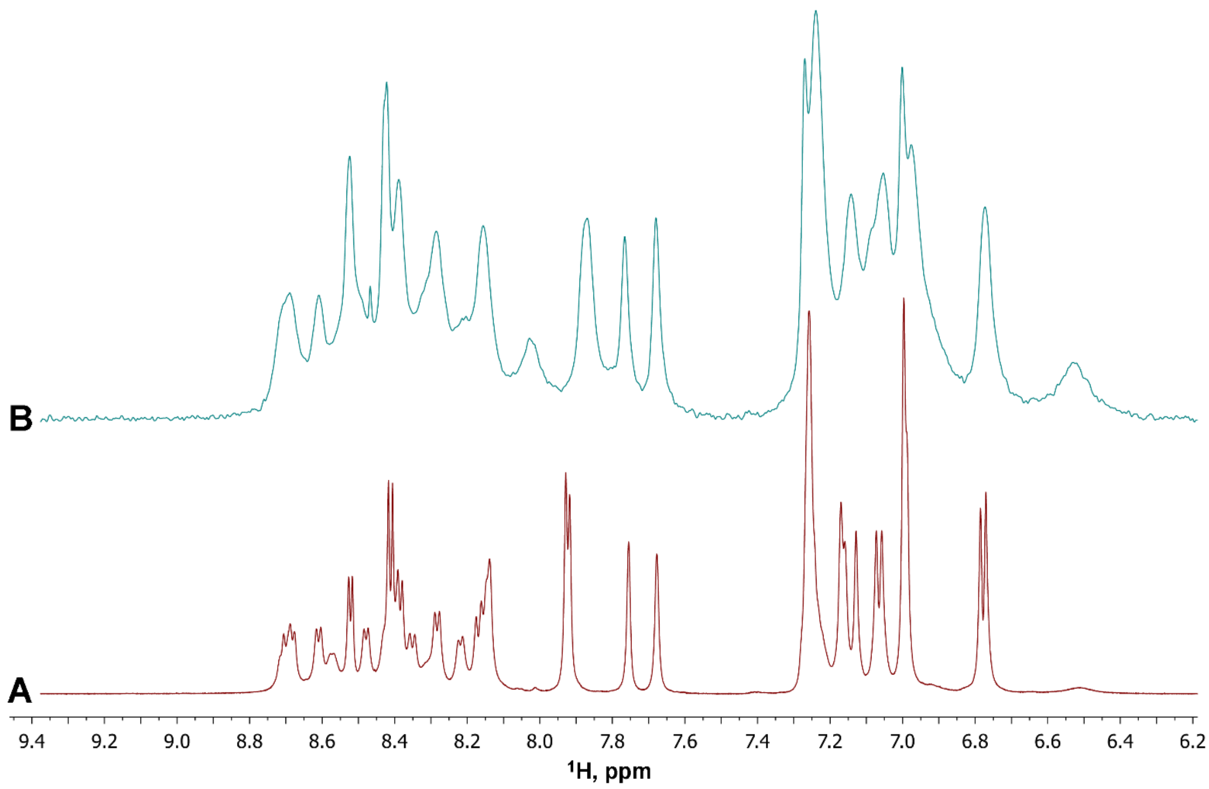

Figure S7. Region of amide and aromatic signals of ^{1}H NMR spectra of isoD7-Aβ_{1-16} (pH 6.8, 0.2 mM) in free state (A) and after addition of equimolar amount of $ZnCl_2$ (B). Spectra were recorded in 90% H_2O/10% D_2O, in the presence of 10 mM bis-tris-d_{19}, pH 6.8, at 283K.

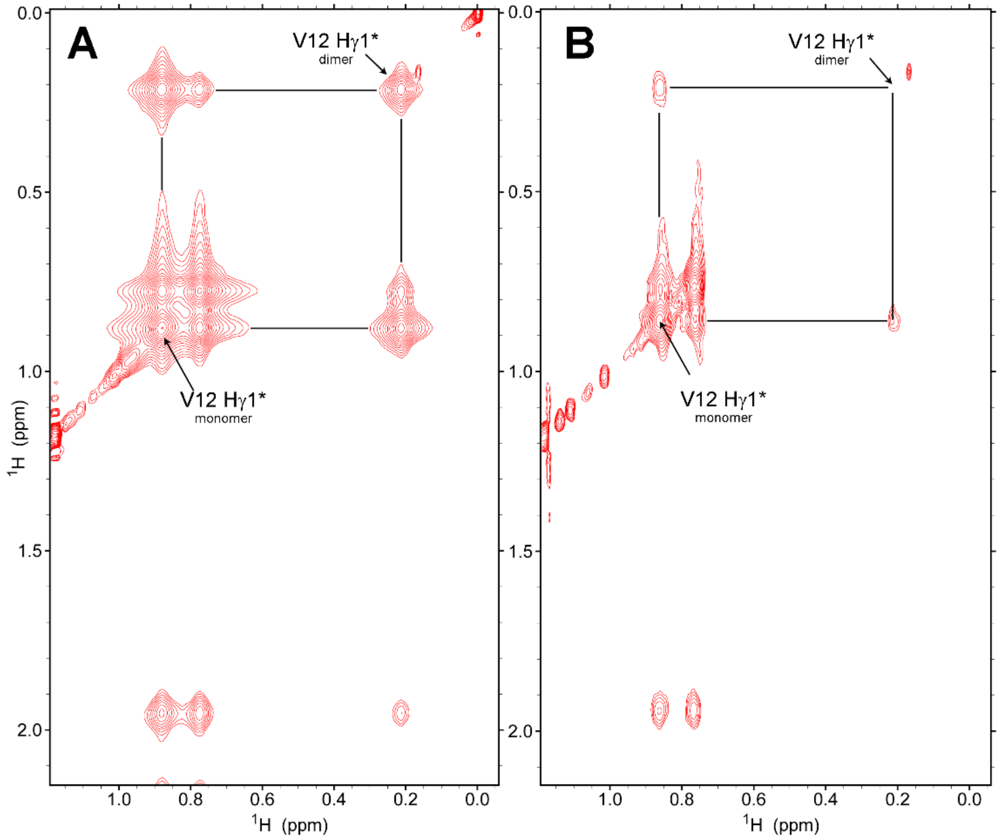

Figure S8. Fragment of NOESY spectra of the ^{Ac}H6R-A$\beta_{1\text{-}16}$ (A) and the ^{Ac}isoD7-A$\beta_{1\text{-}16}$ (B) in the presence of half molar equivalence of Zn^{2+}. Spectra were recorded at 274K in H_2O in the presence of 10 mM bis-Trid-d_{19} buffer, pH 6.8. Concentration of the ^{Ac}H6R-A$\beta_{1\text{-}16}$ is ~2.5 mM, and the ^{Ac}isoD7-A$\beta_{1\text{-}16}$ is ~0.2mM. Correlations between Hγ1* resonances of V12 in monomeric and dimeric forms are shown.

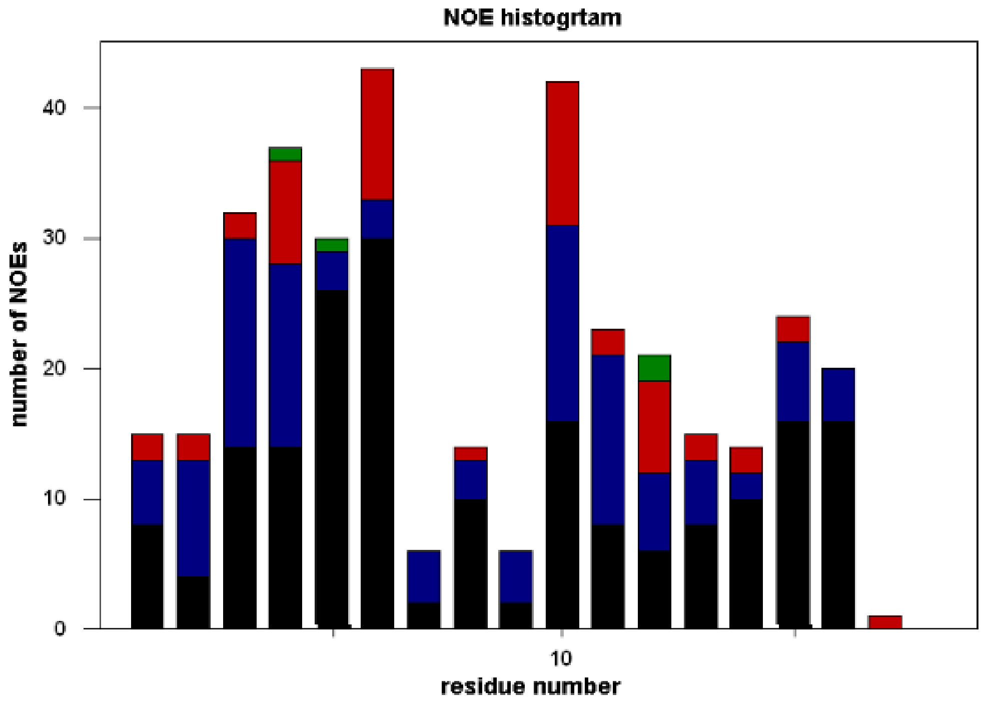

Figure S9. Plot of the number and distribution of NOEs versus the amino acid sequence (one chain) that were used in the structure calculation of the ^{Ac}H6R-A$\beta_{1\text{-}16}$. NOEs are classified as: intra residue (black); sequential (blue, (i-j)=1); medium range (red, 1< (i-j) ≤ 4); long range (green, (i-j)>4).

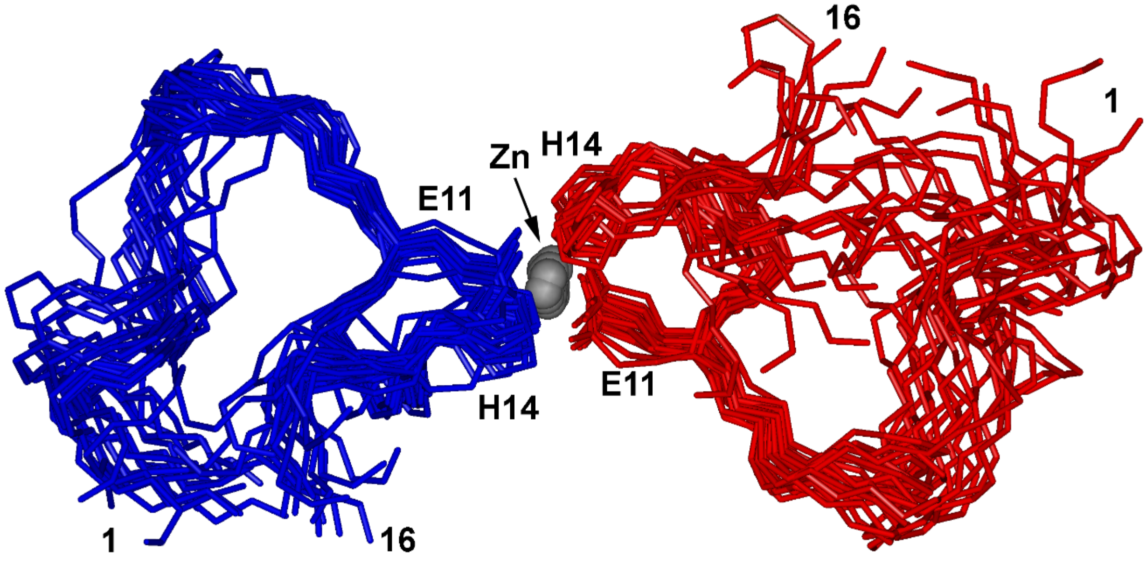

Figure S10. NMR solution structure of the complex of ^{Ac}H6R-A$\beta_{1\text{-}16}$ with Zn^{2+}. Ensemble of the final 20 calculated NMR structures superimposed on the heavy backbone atoms (Cα, N and C) of the residues 2-16 and heavy atoms of the sidechains of the residues E11 and H14. Peptide chains A and B are colored by blue and red correspondingly. Zinc atoms are shown by grey circles.

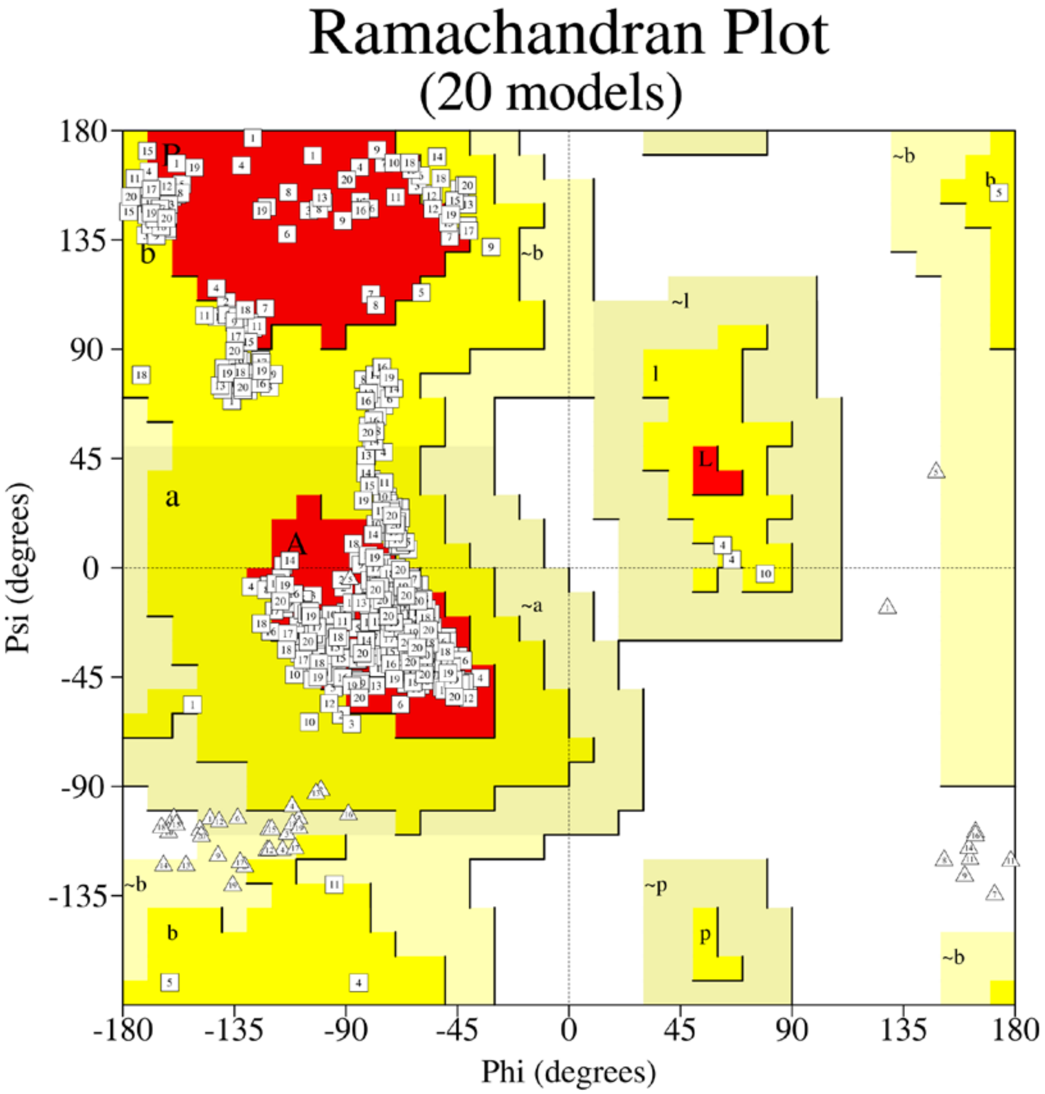

Figure S11. The Ramachandran plot for the final 20 structures of the complex of ^{Ac}H6R-A$\beta_{1\text{-}16}$ with Zn^{2+}. No residues fall in disallowed regions, 73.5% of residues fall in the most favorable regions.

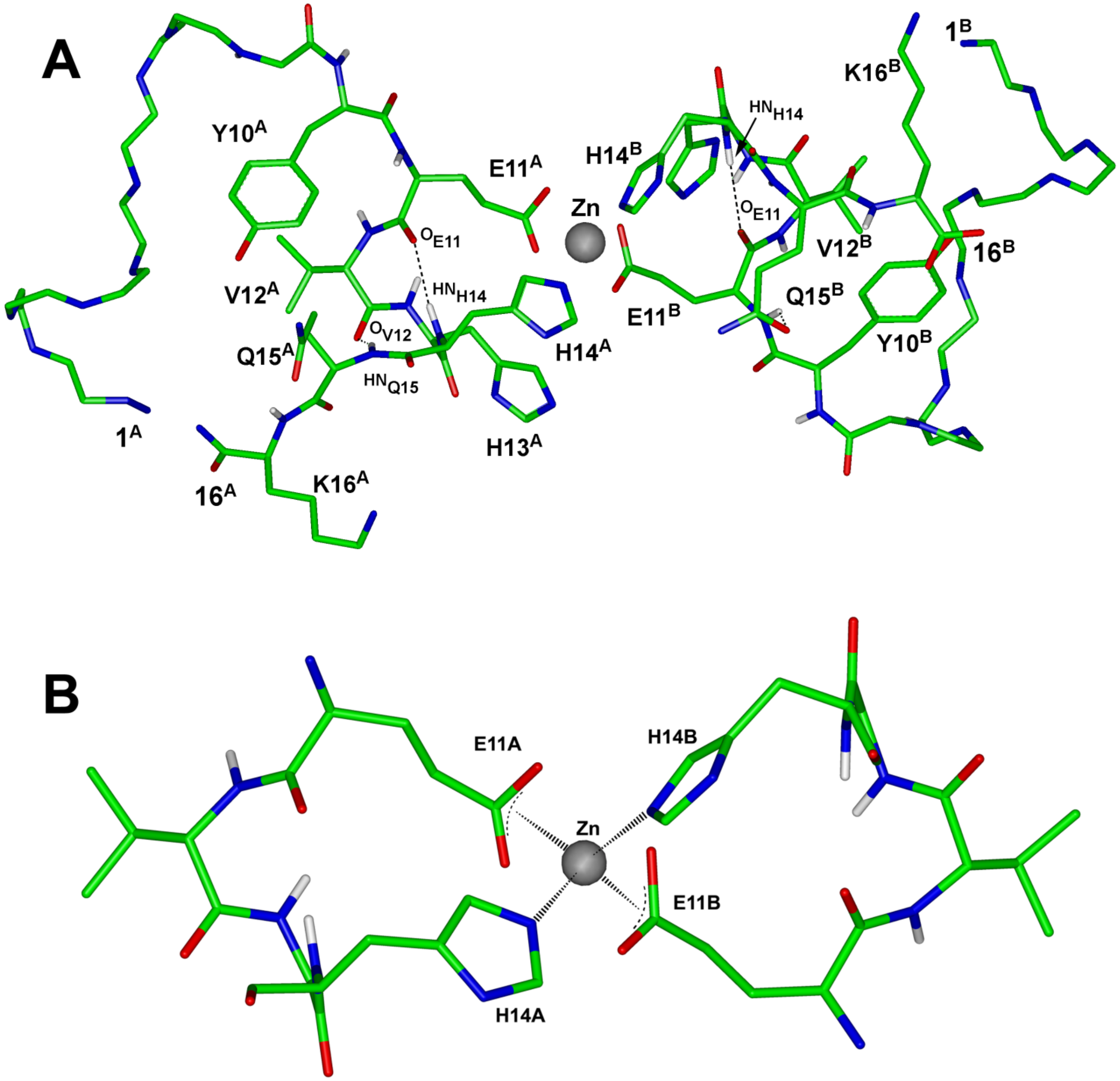

Figure S12. A. Representative structure of the zinc-bridged dimeric peptide ^{Ac}H6R-A$\beta_{1\text{-}16}$. Dotted lines represent contacts of Zn^{2+} ion with the residues E11 and H14 and hydrogen bonds that keep structure of the hydrophobic core of the complex. B. Tetrahedral coordination sphere of zinc ion. Two oxygen atoms of E11 carboxyl group are equivalent in QM/MM optimization protocol.

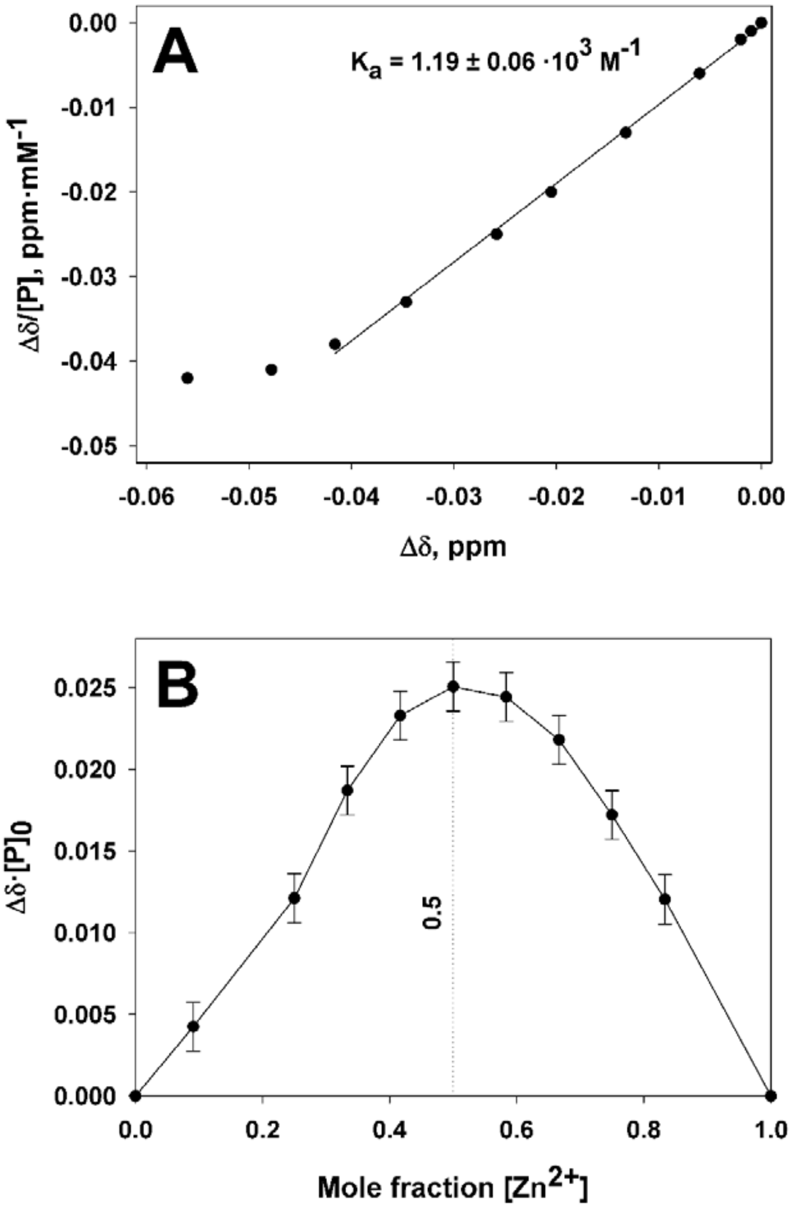

Figure S13. The study of the interaction of zinc ions with the peptide isoD7-A$\beta_{1\text{-}10}$. A. Changes of the chemical shifts of the signals Hβ of residue H6 during titration of the peptide by $ZnCl_2$ shown in Scatchard coordinates. Initial concentration of the peptide was 1.0 mM. Final ratio [Zn^{2+}]/[peptide] = 10. Linearity is disturbed at high concentrations zinc ions (at 5-fold molar excess of Zn^{2+} with respect to the peptide). Shown is the value of binding constant corresponding to the obtained data. B. Results of Zn^{2+} isomolar NMR titration of the peptide isoD7-A$\beta_{1\text{-}10}$. Abscissa represents the mole fraction of $ZnCl_2$, and the ordinate axis shows the product of $\Delta\delta\cdot[P]_0$, where $\Delta\delta$ – chemical shift change and $[P]_0$ – total peptide concentration in the sample. Total concentration of [peptide]+[$ZnCl_2$] in each sample = 1.7 mM. Reference line (dotted) drawn at 0.5 corresponds to 1 : 1 stoichiometry of binding. Isomolar titration experiments were carried out in 90% H_2O/10% D_2O, in the presence of 10 mM bis-tris-d_{19}, pH 6.8 at 283K.

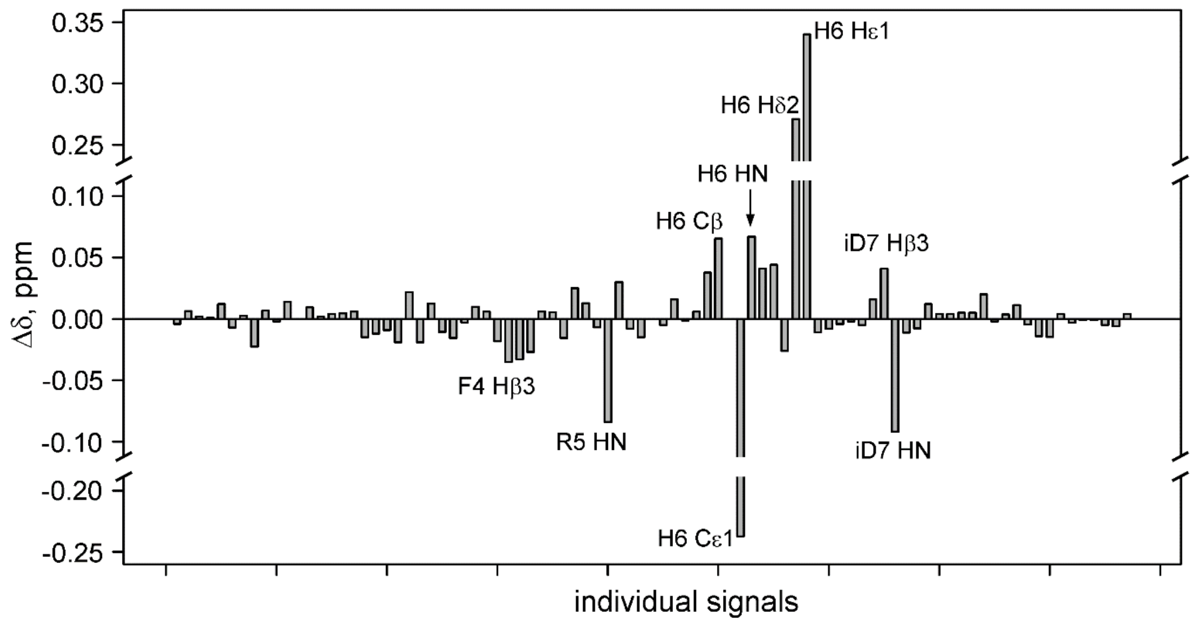

Figure S14. Chemical shift changes of the individual signals of 1.0mM isoD7-Aβ_{1-10} caused by the presence of 3-fold molar excess of Zn^{2+}. Changes in ^{13}C chemical shifts were normalized by a factor of 0.1 for adequate comparison with the ^{1}H data.

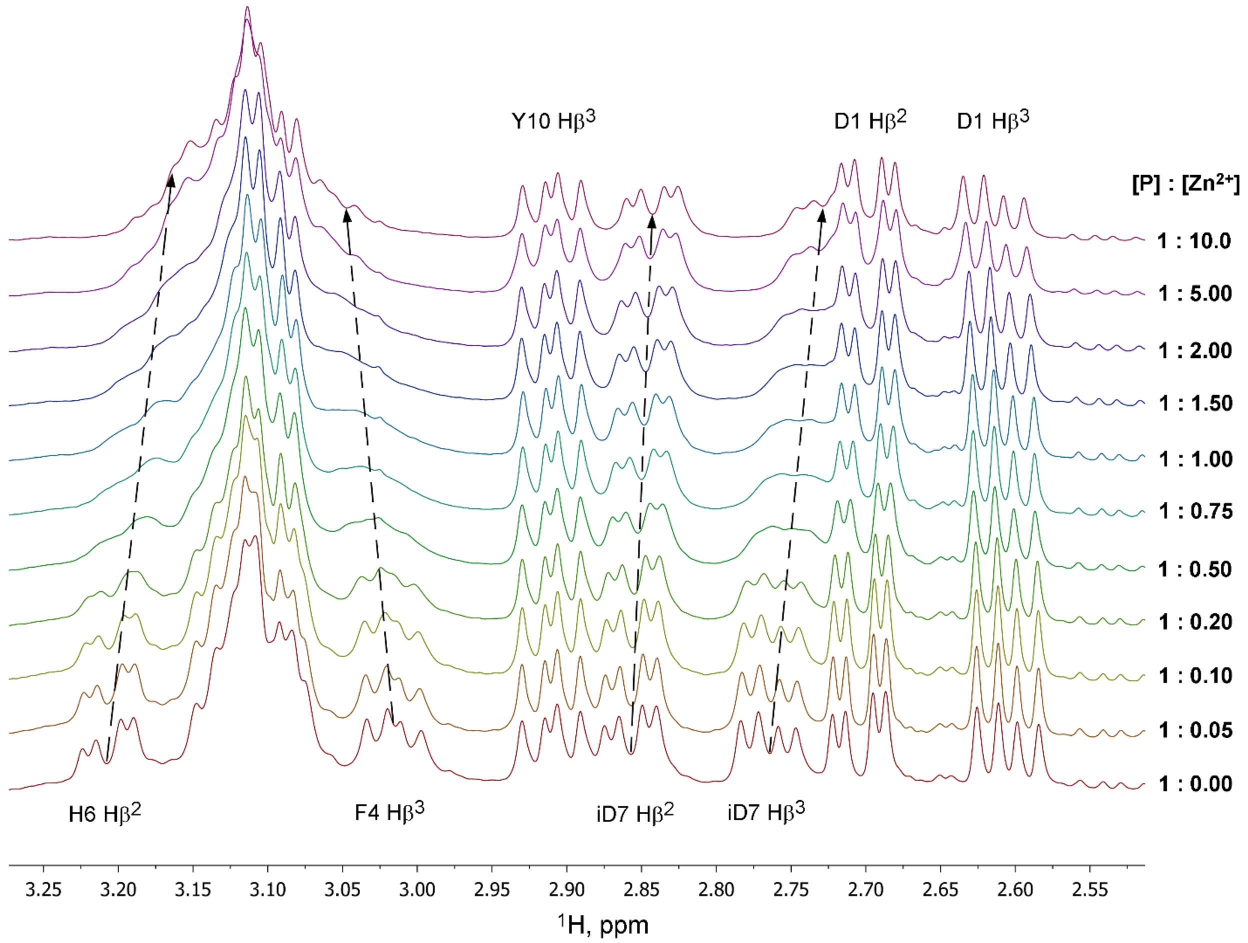

Figure S15. Representative region of the NMR spectra of isoD7-A$\beta_{1\text{-}10}$ at concentration 1.0 mM for the free peptide and series of samples with increased concentration of Zn^{2+}. Spectra were collected in 90% H_2O/10% D_2O in the presence of 10 mM Bis-Tris-d_{19}, at pH 6.8 and 283K. Labels on the right-hand side represent molar ratio of [peptide]:[Zn^{2+}] in each sample. Assignments of the representative resonances are shown.

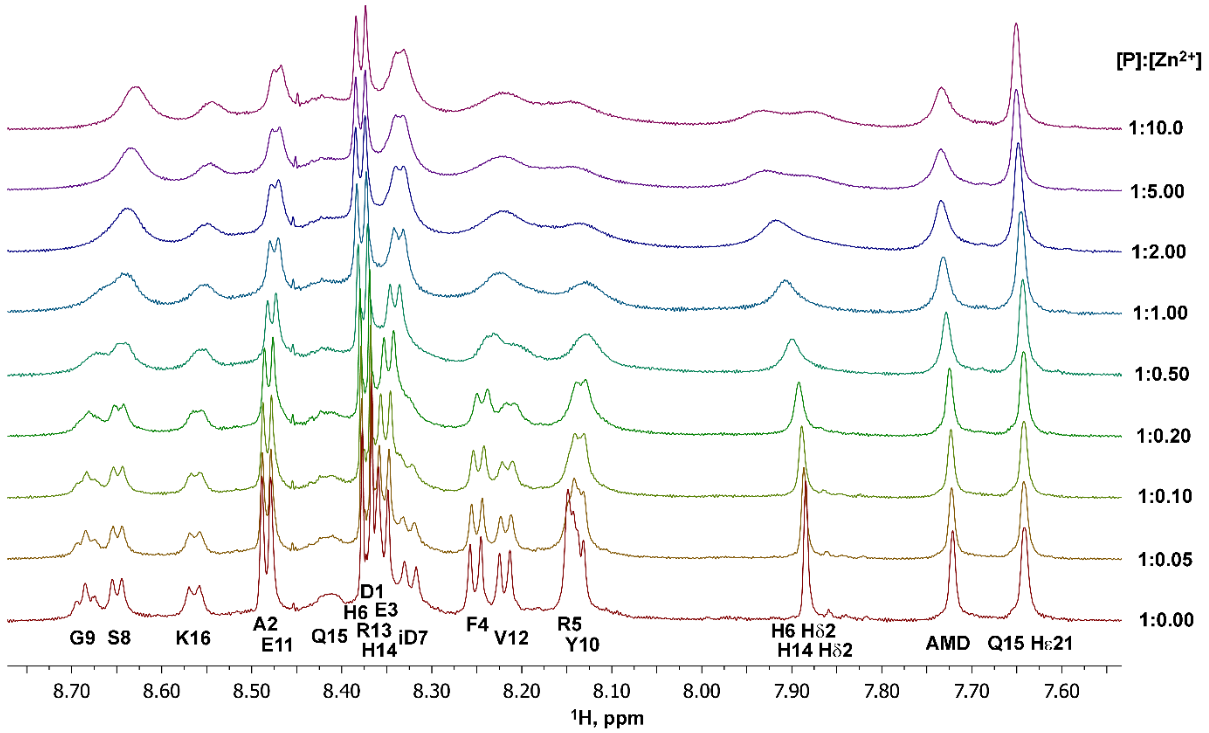

Figure S16. Representative region of the NMR spectra of isoD7,H13R-A$\beta_{1\text{-}16}$ at concentration 1.5 mM for the free peptide and series of samples with increased concentration of Zn^{2+}. Spectra were collected in 90% H_2O/10% D_2O in the presence of 10 mM Bis-Tris-d_{19}, at pH 6.8 and 283K. Labels on the right-hand side represent molar ratio of [peptide]:[Zn^{2+}] in each sample. Signal assignments in free peptide are labeled.

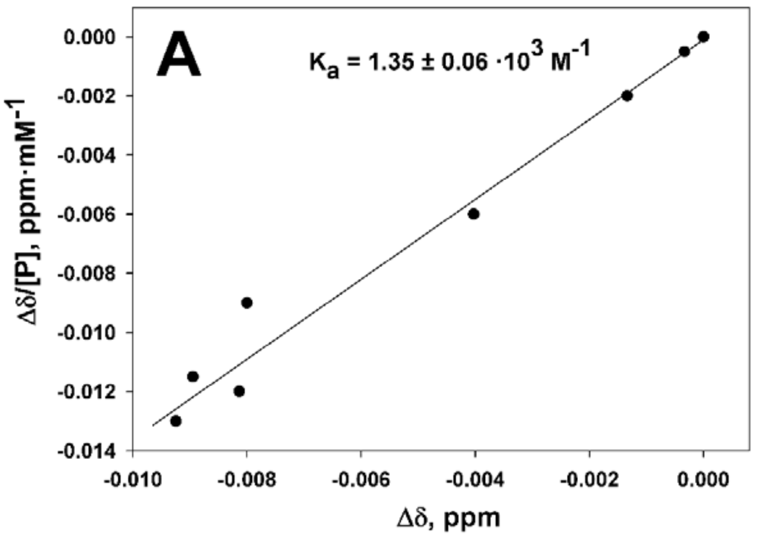

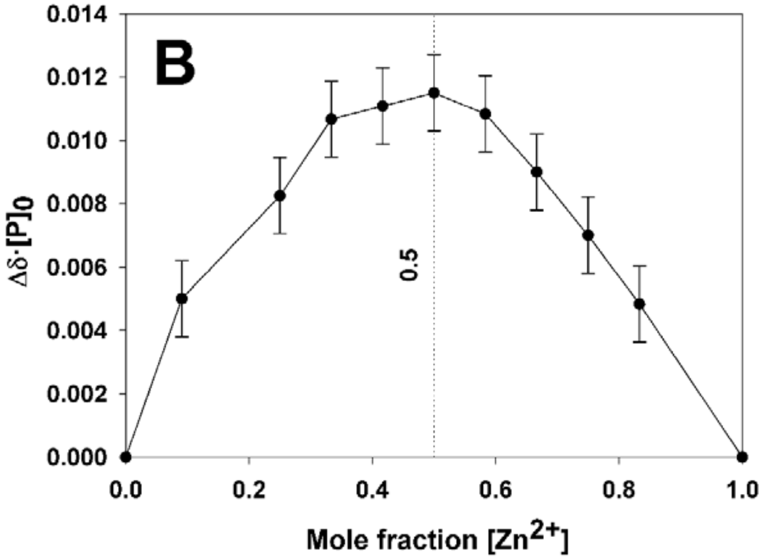

Figure S17. The study of the interaction of zinc ions with the peptide isoD7,H13R-A$\beta_{1\text{-}16}$. A. Changes of the chemical shifts of the signals Hδ* of residue F4 during titration of the peptide by $ZnCl_2$ shown in Scatchard coordinates. Initial concentration of the peptide was 1.5 mM. Final ratio [Zn^{2+}]/[peptide] = 10. Linearity is disturbed at high concentrations zinc ions (at 5-fold molar excess of Zn^{2+} with respect to the peptide). Shown is the value of binding constant corresponding to the obtained data. B. Results of Zn^{2+} isomolar NMR titration of the peptide isoD7-A$\beta_{1\text{-}10}$. Abscissa represents the mole fraction of $ZnCl_2$, and the ordinate axis shows the product of $\Delta\delta\cdot[P]_0$, where Δδ – chemical shift change and $[P]_0$ – total peptide concentration in the sample. Total concentration of [peptide]+[$ZnCl_2$] in each sample = 1.0 mM. Reference line (dotted) drawn at 0.5 corresponds to 1 : 1 stoichiometry of binding. Isomolar titration experiments were carried out in 90% H_2O/10% D_2O, in the presence of 10 mM bis-tris-d_{19}, pH 6.8 at 283K.

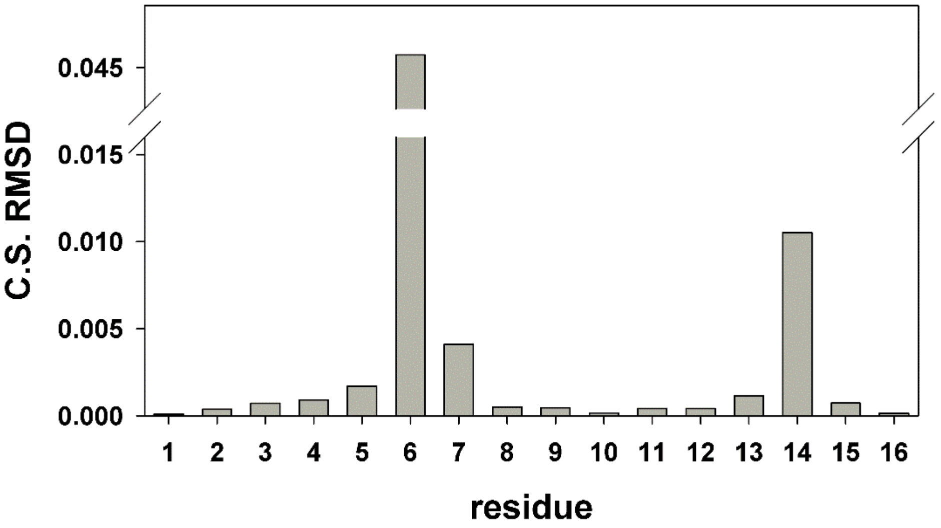

Figure S18. Root-mean-square deviations (RSMD) of the ^{1}H and ^{13}C chemical shifts between the free and zinc-bound states of isoD7,H13R-A$\beta_{1\text{-}16}$. Changes in ^{13}C chemical shifts were normalized by a factor of 0.1 for adequate comparison with the ^{1}H data.

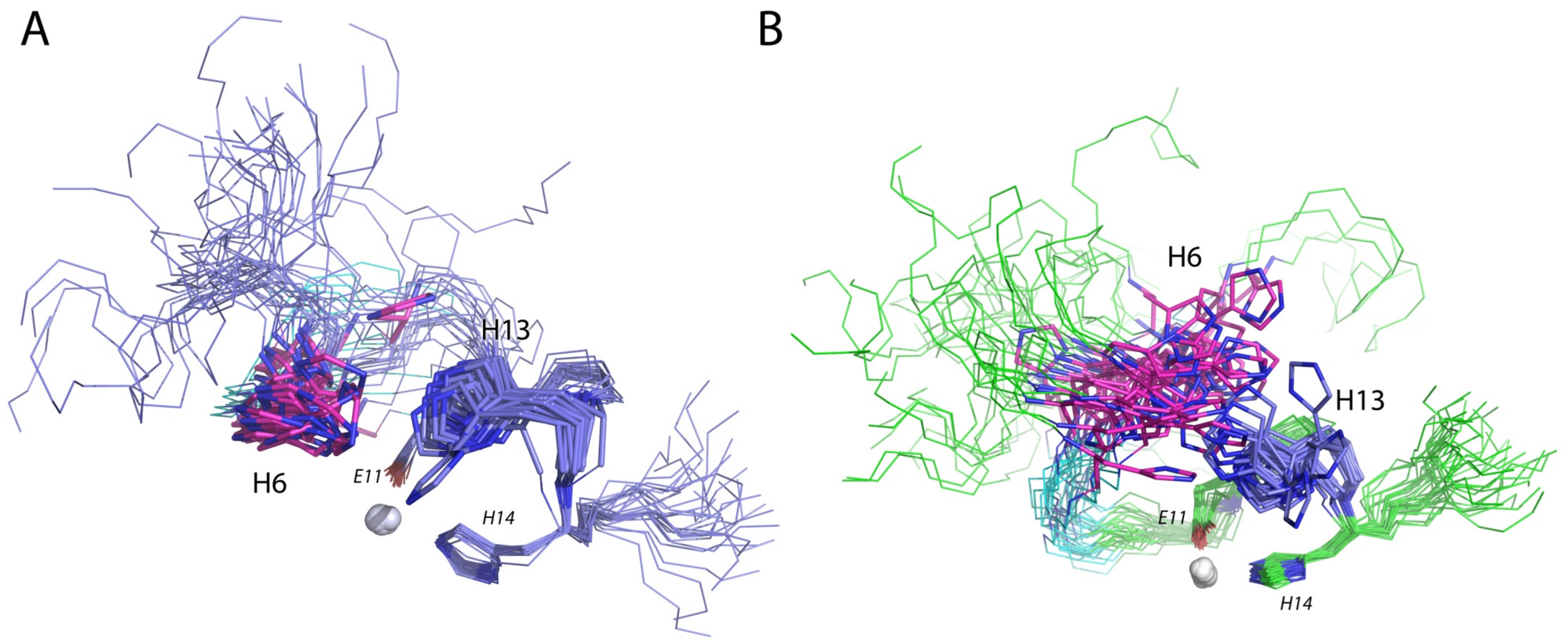

Figure S19. Snapshots from 20 ns restrained molecular dynamic trajectories taken with 1 ns step: (A) $A\beta_{1-16}\cdots A\beta_{1-16}$ and (B) $A\beta_{1-16}\cdots$isoD7-$A\beta_{1-16}$. Residues H6 and H13 are depicted by bold sticks. Zinc ions are shown as grey circles. Backbone atoms of D7, isoD7 and S8 are colored cyan.

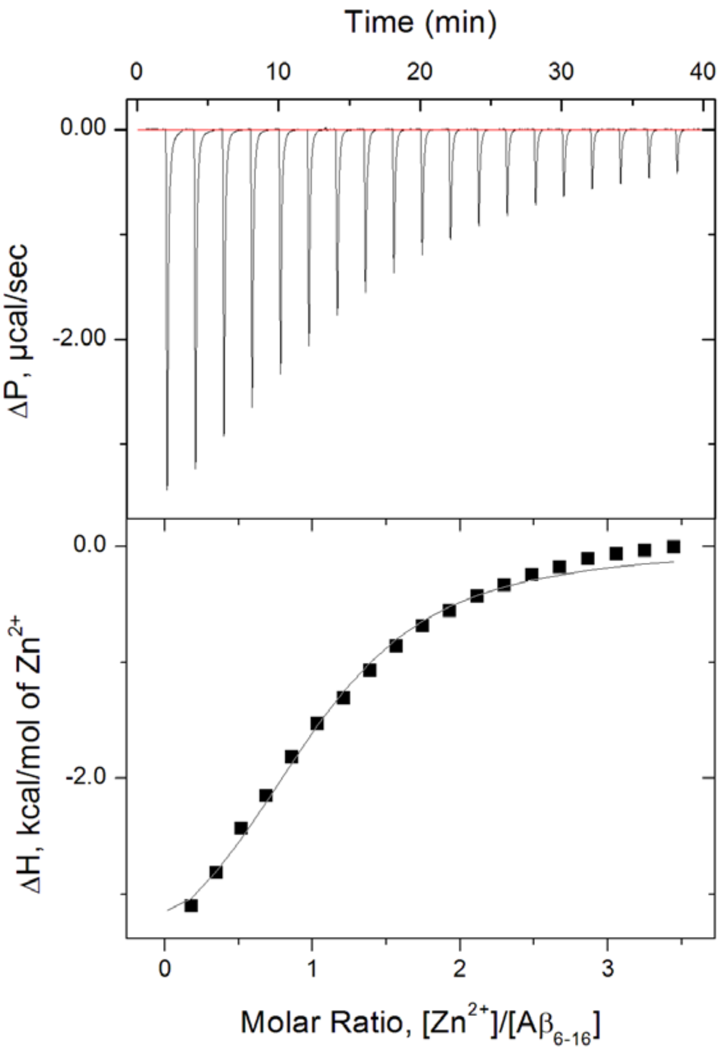

Figure S20. ITC titration curve (upper panel) and the binding isotherm (lower panel) for the zinc (5mM) interaction with $A\beta_{6-16}$ (0.3 mM) at 25˚C in 50 mM Tris buffer, pH 7.3. The thermodynamic parameters of zinc-peptide interactions: $K_a = 1.27 \times 10^4 \pm 0.13\ M^{-1}$; stoichiometry = 1.0; $\Delta H = -4.0 \times 10^3 \pm 0.4$ kcal M^{-1}; $T\Delta S_{298} = 1.6 \pm 0.16$ kcal M^{-1}.

METHODS

Determination of the exchange rates between monomeric and dimeric complexes of ^{Ac}H6R-A$\beta_{1\text{-}16}$.

In order to measure kinetic parameters of the exchange process between monomeric and dimeric forms of the Zn-peptide complex magnetization transfer NMR technique was used (1,2). Modification of the originally proposed by Forsen and Hoffman (1) magnetization-transfer NMR experiment (Fig. S22) involved selective 180° RF pulse with the center on the resonance of Val12 methyl group at 0.88 ppm (Fig. S23, A), a separated from the final reading 90° pulse by the delay, during which inversed magnetization of the resonance A can transfer to the resonance B via exchange mechanism.

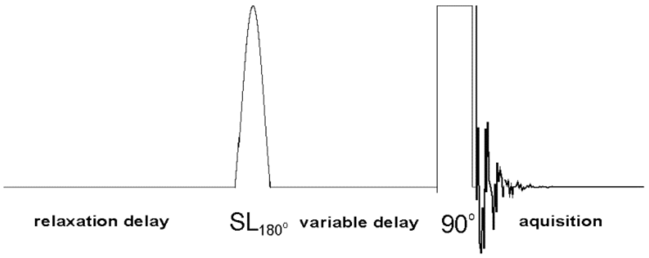

Fig. S22. Pulse sequence for magnetization-transfer NMR study of chemical exchange rates.

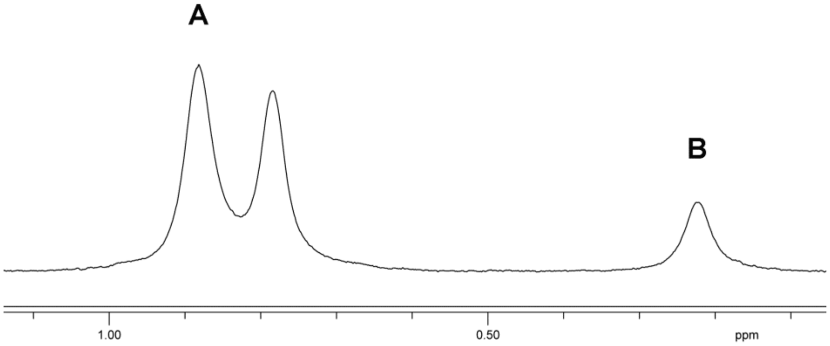

Fig. S23. Fragment of the ^{1}H NMR spectrum of the ^{Ac}H6R-A$\beta_{1\text{-}16}$ with added 0.5 molar equivalent of $ZnCl_2$. Shown are resonances of Val12 methyl groups. Peptide concentration is 2.3 mM. Spectrum recorded in 10 mM Bis-Tris-d_{19} buffer in D_2O (pH 6.8) at 278K. Resonance **A** belongs to monomeric state and resonance **B** belongs to the dimeric complex of the peptide with zinc ion.

For each magnetization transfer delay 1D spectrum was acquired. The following delays were used: 0.00001, 0.001, 0.002, 0.003, 0.004, 0.006, 0.008, 0.01, 0.014, 0.016, 0.018, 0.022, 0.026, 0.03, 0.035, 0.04, 0.05, 0.055, 0.06, 0.065, 0.07, 0.08, 0.1, 0.12, 0.14, 0.16, 0.18, 0.2, 0.25, 0.3, 0.4, 0.6, 0.8, 1.0, 2.0, 5.0 and 10.0 s. For several delays two independent NMR measurements were carried out. Fig.S24 shows changes of the shapes and intensities of the signals in the recorded set of NMR spectra. After the inversion of the resonance A magnetization recovers with the increase of delay between selective and non-selective pulses. At the same time, inversed magnetization from the resonance A transfers to the resonance B thus causing changes of its intensity. Initially intensity of the signal B becomes smaller and then also recovers due to the longitudinal relaxation. Kinetic parameters of the exchange between sites A and B (monomeric and dimeric Zn-peptide complexes correspondingly) can be obtained from the analysis of intensities off the signals A and B.

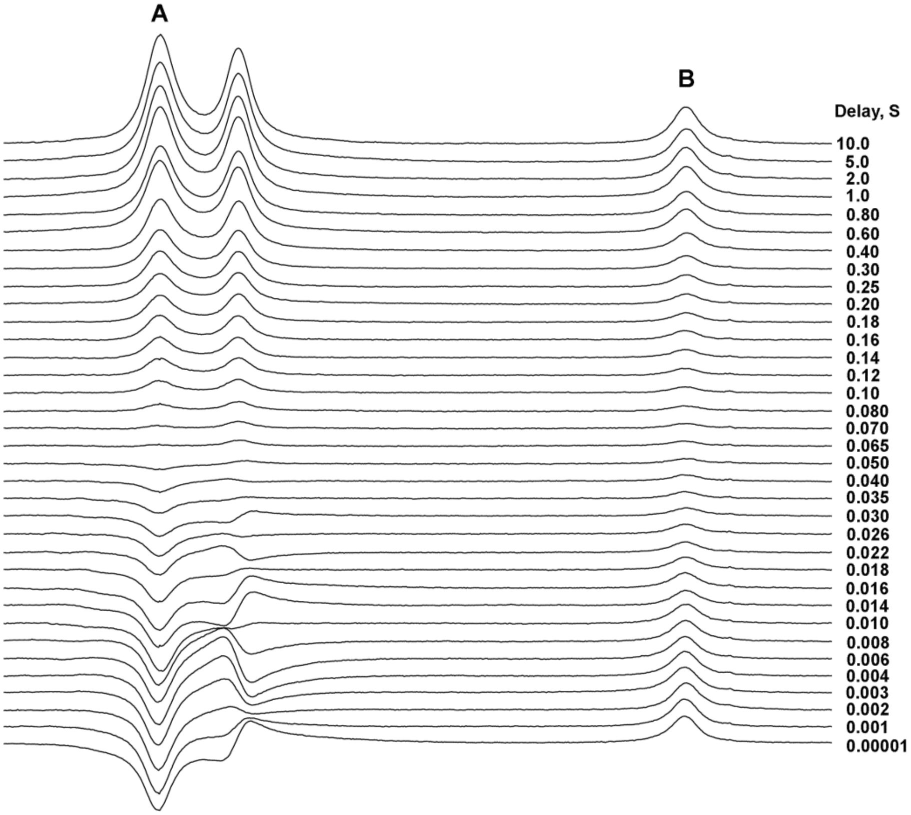

Fig. S24. Series of ^{1}H NMR spectra of the $^{Ac}H6R\text{-}A\beta_{1\text{-}16}$ with added 0.5 molar equivalent of $ZnCl_2$, recorded for various delays between the selective 180° and non-selective 90° pulses. Resonances A and B are marked as explained in Fig. 23 caption.

Theory of magnetization transfer in exchange process was developed by McConnel (3).

$$\boldsymbol{A} \underset{k_B}{\overset{k_A}{\rightleftarrows}} \boldsymbol{B}$$

[1]

For the equilibrium between states A and B change of magnetization M_A and M_B (i.e. their signal intensities) can be described by two-exponential functions.

$$M_A(t) = M_A^{\infty} + C_1 e^{\lambda_1 t} + C_2 e^{\lambda_2 t} \quad [2]$$

$$M_B(t) = M_B^{\infty} + C_3 e^{\lambda_1 t} + C_4 e^{\lambda_2 t} \quad [3]$$

M_A^{∞} and M_B^{∞} - equilibrium intensities of the signals A and B. Coefficients λ_1 and λ_2 depend on the rate constants k_A and k_B and also on longitudinal relaxation rates R_{1A} and R_{1B}:

$$\lambda_1 = \frac{1}{2}\left[-(k_{1A} + k_{1B}) + \sqrt{(k_{1A} - k_{1B})^2 + 4k_A k_B}\right] \quad [4]$$

$$\lambda_2 = \frac{1}{2}\left[-(k_{1A} + k_{1B}) - \sqrt{(k_{1A} - k_{1B})^2 + 4k_A k_B}\right] \quad [5]$$

,where

$$k_{1A} = k_A + R_{1A} \qquad k_{1B} = k_B + R_{1B} \quad [6]$$

In assumption of equal line widths of resonances A and B (26 and 25 Hz correspondingly as measured at 278K) coefficients $C_1 - C_4$ can be written as:

$$C_1 = \frac{[(\lambda_2 + k_{1A})(M_A^{\infty} - M_A^0) - k_B(M_B^{\infty} - M_B^0)]}{(\lambda_1 - \lambda_2)} \quad [7]$$

$$C_2 = \frac{[-(\lambda_1 + k_{1A})(M_A^{\infty} - M_A^0) + k_B(M_B^{\infty} - M_B^0)]}{(\lambda_1 - \lambda_2)} \quad [8]$$

$$C_3 = \frac{[-k_A(M_A^{\infty} - M_A^0) - (\lambda_1 + k_{1A})(M_B^{\infty} - M_B^0)]}{(\lambda_1 - \lambda_2)} \quad [9]$$

$$C_4 = \frac{[k_A(M_A^{\infty} - M_A^0) + (\lambda_2 + k_{1A})(M_B^{\infty} - M_B^0)]}{(\lambda_1 - \lambda_2)} \quad [10]$$

All six parameters (λ_1, λ_2 , M_A^{∞}, $M_B^{\infty}$and $C_1 - C_4$) can be obtained from non-linear fitting of the experimental data using equations 2 and 3. Results of the fitting are shown on Fig. S25.

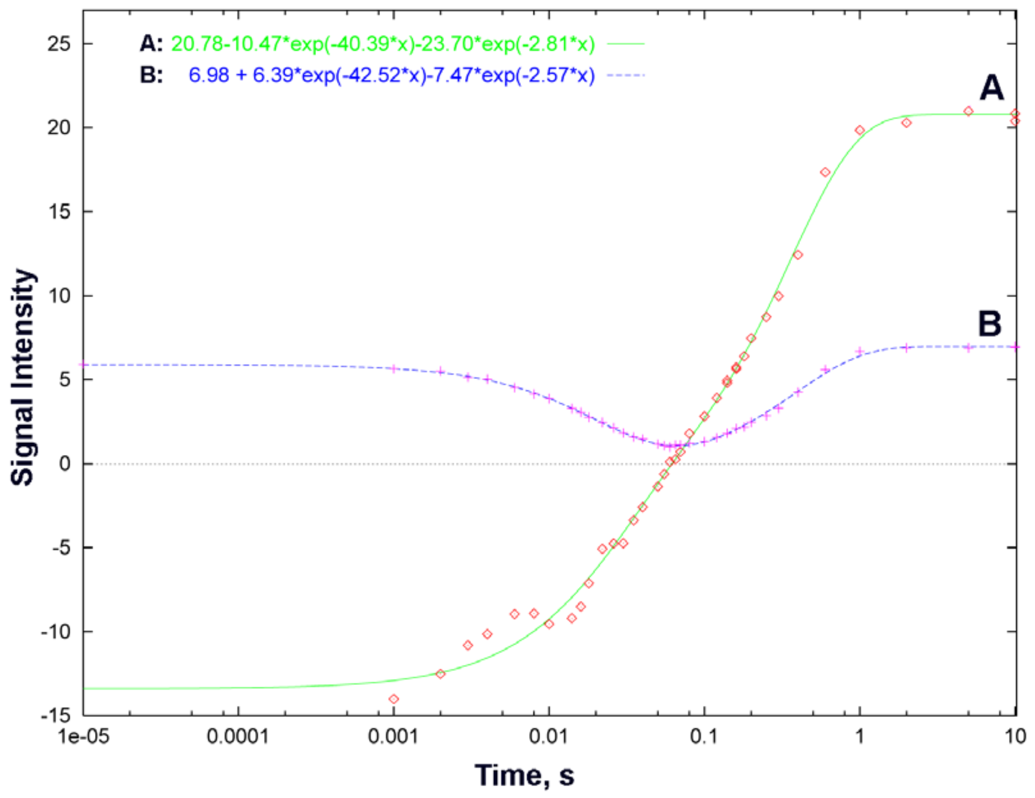

Fig. S25. Results of the non-linear fitting of the changes of resonance A and B. Initially, resonance A was inverted. Solid lines are calculated data and dots are experimentally measured signal intensities. Logarithm scale was used for the time axis.

It should be noted that intensities of the signal A at small values of the delay between selective and non-selective pulses are distorted due to inversion of neighboring signal at 0.79 ppm and additional exchange process between these two signals. More accurate data can therefore be obtained from the fitting of intensities of signal B. However, it is worth noting that parameters λ_1 and λ_2 obtained from the fitting of both sets of intensities (A and B) are nearly identical (within their estimated standard deviations):

Parameter	λ_1	λ_2	C_1	C_2	C_3	C_4	M_A^∞	M_B^∞
Value	-2.57 ± 0.08	-42.52 ± 1.26	-23.71 ± 0.82	-10.47 ± 0.82	-7.47 ± 0.09	6.39 ± 0.09	20.78 ± 0.36	6.98 ± 0.05

From eq. 3 and 4 it can be seen that:

$$\lambda_1 + \lambda_2 = -(k_{1A} + k_{1B}) \qquad [11]$$

From eq. 7 – 10 it is easy to see:

$$C_1 + C_2 \ = -\left(M_A^{\infty} - M_A^0\right) \qquad [12]$$

$$C_3 + C_4 \ = -\left(M_B^{\infty} - M_B^0\right) \qquad [13]$$

In addition to magnetization transfer experiments, measurements of longitudinal relaxation rates R_1 were carried out. It was found that the values of R_1 are nearly identical for the resonances A and B. This information allows simplifying equations 4 and 5. In such case:

$$(k_{1A} - k_{1B}) \ \cong \ (k_A - \ k_B) \ \Rightarrow \ \sqrt{(k_{1A} - k_{1B})^2 + 4k_A k_B} \ \cong \ (k_A + \ k_B)$$

therefore:

$$\lambda_1 = -\frac{1}{2}[(R_{1A} + R_{1B})] \ \cong \ R_{1A} \ \cong \ R_{1B} \qquad [12]$$

$$\lambda_2 = -\frac{1}{2}[(k_{1A} + k_{1B}) + (k_A + k_B)] \qquad [13]$$

$$\lambda_1 - \lambda_2 = (k_A + k_B) \qquad [14]$$

With these approximations, using equations 7-10 (it is possible to use just one of the equations, for example 9 which determines coefficient C_3) it is rather straightforward to obtain values of the rate constants: $k_A = 8.6 \pm 0.6\ s^{-1}$ and $k_B = 31.3 \pm 2.3\ s^{-1}$.

REFERENCES

1. Forsen, S. and R. A. Hoffman. 1963. Study of Moderately Rapid Chemical Exchange Reactions by Means of Nuclear Magnetic Double Resonance. *Journal of Chemical Physics*. 39:2892-2901.

2. Led, J. J. and H. Gesmar. 1982. The Applicability of the Magnetization Transfer NMR Technique to Determine Chemical-Exchange Rates in Extreme Cases - the Importance of Complementary Experiments. *Journal of Magnetic Resonance*. 49:444-463.

3. McConnell, H. M. 1958. Reaction Rates by Nuclear Magnetic Resonance. *Journal of Chemical Physics*. 28:430-431.

Table S1. Chemical shifts (ppm) of the ^{1}H, ^{13}C and ^{15}N signals of free human peptide isoD7-Aβ_{1-16}, measured in 10 mM bis-Trid-d_{19} buffer, pH 6.8.

residue	^{15}N	Cα	Cβ	other C, N	HN	Hα	Hβ	Hγ	other H
**D1**	126.7	54.53	41.28		8.40	4.55	2.70<br>2.61	-	-
**A2**	124.0	52.73	19.04		8.51	4.26	1.37	-	-
**E3**	119.3	56.48	30.09	36.08 (Cγ)	8.37	4.16	1.89	2.18<br>2.09	-
**F4**	120.8	57.52	39.33	131.7 (Cδ*)<br>131.3 (Cε*)<br>129.9 (Cζ)	8.25	4.58	3.09<br>3.01	-	7.19 (Hδ*)<br>7.28 (Hε*)<br>7.26 (Hζ)
**R5**	123.2	55.52	30.93	26.90 (Cγ)<br>43.05 (Cδ)	8.19	4.27	1.71<br>1.63	1.50	3.11 (Hδ*)<br>7.22 (Hε)
**H6**	120.2	56.09	30.50	-	8.44	4.57	3.02	-	
**isoD7**	125.9	54.58	40.44	-	8.44	4.55	2.87<br>2.78	-	-
**S8**	121.6	58.78	63.59	-	8.67	4.39	3.88	-	-
**G9**	110.8	45.19	-	-	8.70	3.91	-	-	-
**Y10**	120.1	57.87	38.76	133.0 (Cδ*)<br>118.0 (Cε*)	8.13	4.54	3.05<br>2.92	-	7.07 (Hδ*)<br>6.77 (Hε*)
**E11**	122.5	56.48	30.25	36.07 (Cγ)	8.45	4.21	1.94<br>1.87	2.21	-
**V12**	121.3	62.66	32.44	20.61 (Cγ1)<br>20.76 (Cγ2)	8.19	3.94	1.94	0.88<br>0.77	-
**H13**	122.0	55.92	30.42	119.5 (Cδ2)<br>137.8 (Cε1)	8.48	4.64	3.06<br>3.13	-	7.09 (Hδ2)<br>8.06 (Hε1)
**H14**	120.8	55.66	30.19	120.4 (Cδ2)	8.44	4.62	3.17<br>3.21	-	7.20 (Hδ2)
**Q15**	122.1	55.77	29.17	33.53 (Cγ)<br>112.9 (Nε2)	8.62	4.31	2.10<br>1.99	2.37	7.67 (Hε21)<br>6.99 (Hε22)
**K16**	123.8	56.11	32.86	24.80 (Cγ)<br>28.87 (Cδ)<br>41.75 (Cε)	8.63	4.26	1.85<br>1.79	1.50<br>1.45	1.69 (Hδ*)<br>3.00 (Hε*)

Table S2. Chemical shifts (ppm) of the ^{1}H and ^{13}C signals of isoD7-Aβ_{1-16}, measured in the presence of twofold molar excess of $ZnCl_2$ in 10 mM bis-Trid-d_{19} buffer, pH 6.8.

residue	Cα	Cβ	other C, N	HN	Hα	Hβ	Hγ	other H
**D1**	54.20	41.05		8.41	4.56	2.70<br>2.61	-	-
**A2**	52.46	18.80		8.51	4.26	1.37	-	-
**E3**	56.29	29.86	35.69 (Cγ)	8.37	4.18	1.89	2.18<br>2.07	-
**F4**	57.30	39.23	131.4(Cδ*)<br>131.0 (Cε*)<br>129.5 (Cζ)	8.27	4.55	3.04<br>3.00	-	7.14 (Hδ*)<br>7.24 (Hε*)<br>7.25 (Hζ)
**R5**	55.11	30.68	26.63 (Cγ)<br>42.91 (Cδ)	8.15	4.29	1.71<br>1.62	1.49	3.10 (Hδ*)
**H6**	56.08	30.11	137.94 (Cε1)	8.37	4.59	3.16<br>3.07	-	
**isoD7**	54.50	40.32	-	8.30	4.53	2.86<br>2.77	-	-
**S8**	58.56	63.47	-	8.67	4.39	3.88	-	-
**G9**	45.05	-	-	8.69	3.90	-	-	-
**Y10**	57.79	38.53	132.8 (Cδ*)<br>117.9 (Cε*)	8.14	4.53	3.04<br>2.90	-	7.05 (Hδ*)<br>6.77 (Hε*)
**E11**			35.70 (Cγ)	8.48	4.21		2.21	-
**V12**	62.43	32.14	20.26 (Cγ1)<br>20.46 (Cγ2)	8.20	3.95	1.95	0.86<br>0.76	-
**H13**	55.42		119.60 (Cδ2)<br>138.22 (Cε1)	8.37	4.56	3.06<br>3.00	-	6.97 (Hδ2)<br>7.87 (Hε1)
**H14**	55.73	30.19	119.60 (Cδ2)<br>138.22 (Cε1)	8.14	4.60	3.09<br>3.01	-	6.97 (Hδ2)<br>7.98 (Hε1)
**Q15**	55.46	28.98	32.34 (Cγ)	8.54	4.31	2.11<br>1.99	2.35	7.66 (Hε21)<br>6.99 (Hε22)
**K16**	55.99	32.67	24.46 (Cγ)<br>28.74 (Cδ)<br>41.70 (Cε)	8.60	4.26	1.85<br>1.78	1.45	1.68 (Hδ*)<br>2.99 (Hε*)

Table S3. Chemical shifts (ppm) of the ^{1}H, ^{13}C and ^{15}N signals of free peptide isoD7-H13R-A$\beta_{1\text{-}16}$, measured in 10 mM bis-Trid-d_{19} buffer, pH 6.8.

residue	^{15}N	Cα	Cβ	other C, N	HN	Hα	Hβ	Hγ	other H
**D1**	126.8	54.81	41.43		8.41	4.55	2.70<br>2.61	-	-
**A2**	124.1	52.95	19.09		8.52	4.25	1.38	-	-
**E3**	119.5	56.75	30.38	36.10 (Cγ)	8.39	4.17	1.90	2.18<br>2.06	-
**F4**	121.2	57.56	39.60	131.7 (Cδ*)<br>131.4 (Cε*)<br>129.7 (Cζ)	8.28	4.56	3.07<br>3.01	-	7.17 (Hδ*)<br>7.27 (Hε*)<br>7.25 (Hζ)
**R5**	123.5	56.04	31.05	27.05 (Cγ)<br>43.47 (Cδ)	8.17	4.28	1.71<br>1.63	1.50	3.10 (Hδ*)<br>7.23 (Hε)
**H6**	120.8	56.33	30.39	120.5 (Cδ2)<br>137.5 (Cε1)	8.41	4.60	3.18<br>3.12	-	7.14 (Hδ2)<br>8.17 (Hε1)
**isoD7**	125.9	55.09	40.61	-	8.37	4.54	2.86<br>2.78	-	-
**S8**	121.8	59.16	63.81	-	8.69	4.39	3.90<br>3.87	-	-
**G9**	111.0	45.43	-	-	8.71	3.91	-	-	-
**Y10**	120.5	57.46	39.03	133.2 (Cδ*)<br>118.1 (Cε*)	8.17	4.50	3.06<br>2.95	-	7.08 (Hδ*)<br>6.77 (Hε*)
**E11**	122.4	56.82	29.96	36.25 (Cγ)	8.51	4.22	1.99<br>1.92	2.23	-
**V12**	121.8	62.99	32.45	20.94 (Cγ1)<br>21.05 (Cγ2)	8.26	3.98	2.03	0.96<br>0.87	-
**R13**	124.2	56.12	30.91	27.28 (Cγ)<br>43.34 (Cδ)	8.39	4.28	1.73	1.54<br>1.50	3.13 (Hδ*)<br>7.26 (Hε)
**H14**	120.7	56.19	30.72	119.8 (Cδ2)<br>138.1 (Cε1)	8.39	4.61	3.12<br>3.01	-	7.01 (Hδ2)<br>7.91 (Hε1)
**Q15**	122.0	56.02	29.53	33.72 (Cγ)<br>112.9 (Nε2)	8.45	4.31	2.11<br>2.00	2.36	7.60 (Hε21)<br>7.00 (Hε22)
**K16**	123.6	56.59	33.07	24.91 (Cγ)<br>29.07 (Cδ)<br>42.11 (Cε)	8.61	4.26	1.86<br>1.79	1.49<br>1.45	1.70 (Hδ*)<br>3.00 (Hε*)

Table S4. Chemical shifts (ppm) of the ^{1}H, ^{13}C and ^{15}N signals of ^{iso}D7-H13R-A$\beta_{1-16,}$ measured in the presence of twofold molar excess of $ZnCl_2$ in 10 mM bis-Trid-d_{19} buffer, pH 6.8.

residue	^{15}N	Cα	Cβ	other C, N	HN	Hα	Hβ	Hγ	other H
**D1**	126.8	55.35	41.86		8.406	4.554	2.688<br>2.615	-	-
**A2**	124.0	53.29	19.59		8.518	4.250	1.376	-	-
**E3**	119.3	57.13	30.75	36.58 (Cγ)	8.372	4.188	1.900	2.178<br>2.082	-
**F4**	121.1	57.71	39.98	132.2(Cδ*)<br>131.9 (Cε*)<br>130.3 (Cζ)	8.283	4.569	3.096<br>3.039	-	7.20 (Hδ*)<br>7.29 (Hε*)<br>7.26 (Hζ)
**R5**	NA	56.89	31.37	27.60 (Cγ)<br>43.81 (Cδ)	8.251	4.247	1.717<br>1.641	1.484	3.10 (Hδ*)
**H6**	121.0	NA	30.24	127.4 (Cδ2)<br>139.8 (Cε1)	8.357	4.577	3.128<br>3.023	-	6.88 (Hδ2)<br>7.86 (Hε1)
**isoD7**	NA	55.00	41.18	-	8.356	4.545	2.854<br>2.728	-	-
**S8**	121.6	59.59	64.20	-	8.683	4.382	3.913<br>3.868	-	-
**G9**	111.0	45.90	-	-	8.668	3.920	-	-	-
**Y10**	120.5	NA	39.56	133.6 (Cδ*)<br>118.6 (Cε*)	8.181	4.521	3.040<br>2.934	-	7.06 (Hδ*)<br>6.77 (Hε*)
**E11**	122.5	57.36	30.52	36.58 (Cγ)	8.512	4.238	1.973<br>1.920	2.228	-
**V12**	121.8	63.70	32.87	21.54 (Cγ1)<br>21.54 (Cγ2)	8.270	3.980	2.036	0.966<br>0.877	-
**R13**	NA	56.42	31.23	27.75 (Cγ)<br>43.74 (Cδ)	8.405	4.263	1.726	-	3.14 (Hδ*)
**H14**	NA	57.03	31.07	120.0 (Cδ2)<br>139.8 (Cε1)	8.413	4.604	3.133<br>3.021	-	7.00 (Hδ2)<br>7.85 (Hε1)
**Q15**	1	56.63	30.10	32.21 (Cγ)	8.459	4.279	2.114<br>1.996	2.371	7.68<br>(Hε21)<br>6.99<br>(Hε22)<br>112.9 (Nε)
**K16**	123.6	57.15	33.51	25.45 (Cγ)<br>29.66 (Cδ)<br>42.56 (Cε)	8.584	4.242	1.849<br>1.799	1.470	1.70 (Hδ*)<br>3.00 (Hε*)

Table S5. Chemical shifts (ppm) of the ^{1}H, ^{13}C and ^{15}N signals of free peptide isoD7-Aβ_{1-10}, measured in 10 mM bis-Trid-d_{19} buffer, pH 6.8.

residue	^{15}N	Cα	Cβ	other C, N	HN	Hα	Hβ	Hγ	other H
**D1**	126.8	58.02	41.58		8.41	4.55	2.70<br>2.61	-	-
**A2**	124.1	53.01	19.35		8.52	4.26	1.38	-	-
**E3**	119.5	56.89	30.40	36.35 (Cγ)	8.38	4.16	1.90	2.18<br>2.07	-
**F4**	121.2	57.87	39.66	131.9 (Cδ*)<br>131.6 (Cε*)<br>130.1 (Cζ)	8.27	4.56	3.08<br>3.02	-	7.18 (Hδ*)<br>7.28 (Hε*)<br>7.28 (Hζ)
**R5**	123.6	55.76	31.26	27.20 (Cγ)<br>43.43 (Cδ)	8.16	4.28	1.72<br>1.64	1.50	3.11 (Hδ*)<br>7.20 (Hε)
**H6**	120.9	56.08	30.43	120.5 (Cδ2)<br>137.6 (Cε1)	8.43	4.59	3.20<br>3.13	-	7.16 (Hδ2)<br>8.21 (Hε1)
**isoD7**	126.0	54.81	40.77	-	8.33	4.52	2.85<br>2.77	-	-
**S8**	121.5	59.08	63.93	-	8.63	4.37	3.87	-	-
**G9**	110.9	45.49	-	-	8.63	3.87	-	-	-
**Y10**	120.8	55.01	39.14	133.3 (Cδ*)<br>118.2 (Cε*)	8.17	4.53	3.10<br>2.91	-	7.12 (Hδ*)<br>6.82 (Hε*)

Table S6. Chemical shifts (ppm) of the ^{1}H, ^{13}C and ^{15}N signals of the peptide isoD7-A$\beta_{1\text{-}10}$, measured in the presence of twofold molar excess of $ZnCl_2$ in 10 mM bis-Trid-d_{19} buffer, pH 6.8.

residue	^{15}N	Cα	Cβ	other C, N	HN	Hα	Hβ	Hγ	other H
**D1**	126.8	58.06	41.51		8.40	4.54	2.69<br>2.62	-	-
**A2**	124.0	53.23	19.28		8.53	4.24	1.38	-	-
**E3**	119.3	56.87	30.36	36.30 (Cγ)	8.37	4.18	1.91	2.19<br>2.09	-
**F4**	121.2	58.06	39.54	132.0 (Cδ*)<br>131.7Cε*)<br>130.1 (Cζ)	8.26	4.55	3.10<br>3.05	-	7.21 (Hδ*)<br>7.30 (Hε*)<br>7.27 (Hζ)
**R5**	123.7	55.92	31.02	27.27 (Cγ)<br>43.30 (Cδ)	8.25	4.25	1.73<br>1.65	1.49	3.12 (Hδ*)<br>7.20 (Hε)
**H6**	121.0	55.70	29.77	140.0 (Cε1)	8.37	4.55	3.15	-	6.89 (Hδ2)<br>7.87 (Hε1)
**isoD7**	126.9	54.89	40.81	-	8.33	4.53	2.84<br>2.73	-	-
**S8**	121.5	59.19	64.00	-	8.62	4.36	3.87	-	-
**G9**	110.8	45.44	-	-	8.61	3.88	-	-	-
**Y10**	120.7	54.89	39.18	133.5 (Cδ*)<br>118.4 (Cε*)	8.17	4.54	3.10<br>2.91	-	7.13 (Hδ*)<br>6.83 (Hε*)

Table S7. Chemical shifts (ppm) of the ^{1}H, ^{13}C and ^{15}N signals of free peptide $A\beta_{6-16}$, measured in 10 mM bis-Trid-d_{19} buffer, pH 6.8.

residue	^{15}N	Cα	Cβ	other C, N	HN	Hα	Hβ	Hγ	other H
**H6**	125.3	55.98	30.41	119.7 (Cδ2)<br>137.7 (Cε1)	8.46	4.59	3.10<br>3.04	-	7.06 (Hδ2)<br>8.02 (Hε1)
**D7**	121.8	53.88	40.99	-	8.65	4.54	2.70<br>2.65	-	-
**S8**	117.0	58.98	63.56	-	8.56	4.39	3.93<br>3.88	-	-
**G9**	110.8	45.22	-	-	8.64	3.92	-	-	-
**Y10**	120.1	58.16	38.66	133.0 (Cδ*)<br>118.0 (Cε*)	8.07	4.52	3.04<br>2.96	-	7.07 (Hδ*)<br>6.79 (Hε*)
**E11**	122.6	56.45	30.14	36.07 (Cγ)	8.47	4.20	1.93<br>1.85	2.193	-
**V12**	121.3	62.90	32.34	20.67 (Cγ1)<br>20.72 (Cγ2)	8.20	3.93	1.95	0.89<br>0.78	-
**H13**	122.2	55.94	30.29	119.5 (Cδ2)<br>137.9 (Cε1)	8.40	4.62	3.05	-	7.01 (Hδ2)<br>7.94 (Hε1)
**H14**	121.1	56.23	30.29	119.5 (Cδ2)<br>137.9 (Cε1)	8.30	4.59	3.10<br>3.03	-	7.01 (Hδ2)<br>7.94 (Hε1)
**Q15**	122.0	55.75	29.15	33.50 (Cγ)<br>113.0 (Nε2)	8.57	4.30	2.11<br>1.99	2.31	7.68 (Hε21)<br>7.00 (Hε22)
**K16**	123.7	56.21	32.86	24.84 (Cγ)<br>28.97 (Cδ)<br>41.92 (Cε)	8.61	4.26	1.86<br>1.79	1.47	1.70 (Hδ*)<br>3.00 (Hε*)
